# High-resolution structures of a thermophilic eukaryotic 80S ribosome reveal atomistic details of translocation

**DOI:** 10.1038/s41467-022-27967-9

**Published:** 2022-01-25

**Authors:** Miglė Kišonaitė, Klemens Wild, Karine Lapouge, Thomas Ruppert, Irmgard Sinning

**Affiliations:** 1 Biochemiezentrum der Universität Heidelberg (BZH), INF328, D-69120 Heidelberg, Germany; 2 Zentrum für Molekulare Biologie der Universität Heidelberg, INF282, D-69120 Heidelberg, Germany

**Keywords:** Structural biology, Cryoelectron microscopy

## Abstract

Ribosomes are complex and highly conserved ribonucleoprotein assemblies catalyzing protein biosynthesis in every organism. Here we present high-resolution cryo-EM structures of the 80S ribosome from a thermophilic fungus in two rotational states, which due to increased 80S stability provide a number of mechanistic details of eukaryotic translation. We identify a universally conserved ‘nested base-triple knot’ in the 26S rRNA at the polypeptide tunnel exit with a bulged-out nucleotide that likely serves as an adaptable element for nascent chain containment and handover. We visualize the structure and dynamics of the ribosome protective factor Stm1 upon ribosomal 40S head swiveling. We describe the structural impact of a unique and essential m^1^acp^3^ Ψ 18S rRNA hyper-modification embracing the anticodon wobble-position for eukaryotic tRNA and mRNA translocation. We complete the eEF2-GTPase switch cycle describing the GDP-bound post-hydrolysis state. Taken together, our data and their integration into the structural landscape of 80S ribosomes furthers our understanding of protein biogenesis.

## Abstract (web summary)

High-resolution cryo-EM structures of a thermophilic 80S ribosome in two rotational states in complex with the protective factor Stm1, eEF2, and nascent chains are presented, providing insights into rRNA modifications and eukaryotic translation.

## Introduction

Ribosomes are complex macromolecular assemblies consisting of ribosomal proteins (RPs) and ribosomal RNA (rRNA), which catalyze protein synthesis in all cells. In the past decades, huge advances in understanding eukaryotic protein biosynthesis have been made with key contributions from structure determination of vacant, mRNA, tRNA, and different associated factors containing ribosomes from various organisms^1–5^. For the late phase of translation, in order to complete the elongation cycle after peptide-bond formation, the deacylated P-site tRNA needs to be translocated to the E-site, and the mRNA accordingly one codon forward.

Translocation is induced by rotation of the small ribosomal 40S subunit in respect to the large 60S subunit, and rotation is complemented by swiveling of the 40S head perpendicular to the rotation axis of the 40S body. During translocation, in both prokaryotes and eukaryotes, the tRNAs move from the non-rotated A/A P/P PRE state, via various A/P P/E rotated hybrid states, to the translocated P/P E/E POST state^6–10^.

In a classical mechanistic view of translation, mostly derived from the bacterial system, the whole machinery translocates the tRNAs and attached mRNA without frame shifting, and stops in the forward position (induced by a *doorstop* or *pawl*) that keeps the tRNA_2_•mRNA module in the translocated POST state and prevents a backward movement of the tRNAs^11–14^. The mRNA translocation step is physically driven by correlated tRNA movements guided by the framework of the ribosome that by itself acts as a ‘Brownian-ratchet’^6,15–17^. Translocation in eukaryotes is catalyzed by elongation factor eEF2^18–20^. Like its bacterial counterpart EF-G, the eEF2-GTPase is a multidomain protein that undergoes large conformational changes upon ribosome binding, PRE-POST rotation, and finally upon GTP hydrolysis^12,21–23^. It stretches from the decoding center (DC) in the heart of the ribosome via the factor-binding site (FBS) to the GTPase-activating center (GAC) at the sarcin-ricin loop (SRL) of the 60S subunit. Recent years provided first insights into the eEF2 mechanism depending on the unique diphthamide (Dph) modification of a histidine in domain IV at the very tip of eEF2^24,25^. The precision of the translation process is crucial for all organisms, but despite the plethora of ribosomal research, our atomistic understanding is far from being complete as it requires high-resolution structural data for all functional intermediates, which only now become amenable.

Here we focus on late intermediates of translation using the thermophilic fungus *Chaetomium thermophilum (C. thermophilum*, *Ct)*^26,27^. Proteins and complexes from this fungus have proved beneficial for structural analyses, e.g. of proteins involved in ribosome biogenesis and assembly^28,29^. Thermophiles had an immense impact in ribosome research due to their enhanced stability allowing for structure determination at relatively high resolution. However, while this powerful tool was mainly used for prokaryotic ribosomes, its exploitation for eukaryotes lags far behind. Using isolated 80S ribosomes from *C. thermophilum*, we present single-particle cryo-electron microscopy (cryo-EM) structures of an idle POST state ribosome and of a hybrid pe/E tRNA translocation-intermediate (TI)-POST state (0° rotation, 7° swiveling) bound to eEF2-GDP-Mg^2+^. An additional factor present in both states, although no starvation protocol has been applied during cell growth, is the protein Stm1, both a protective^30^ and translation suppressing factor^31^. Stm1 is described here in detail, and we find it also bound to the non-rotated (and non-swiveled) POST state, which was not observed so far. Despite treatment with the antibiotic puromycin that is commonly used for forcing nascent chain (NC) release, NCs are present in both ribosomal states. We define a universal and adaptable RNA tertiary motif narrowing the very end of the polypeptide tunnel exit. Furthermore, we observe numerous chemical modifications of the rRNA and ribosomal proteins including N-terminal acetylation and hundreds of ions modelled as magnesium. We describe details of the Dph modification of eEF2 bound to GDP-Mg^2+^ in contact with Stm1 in the ribosomal A-site. The invariant m^1^acp^3^Ψ 18S rRNA modification in the 40S head serves as part of a ‘*wobble-seal*’ and seems involved in in-frame shifting during translocation. Finally, we detail the eEF2-GTPase switch cycle, which until now was not fully understood due to the lack of high-resolution structures of all eEF2 states in context of the ribosome.

## Results

### Overall structure of *C. thermophilum* 80S ribosomes

**Fig. 1:**
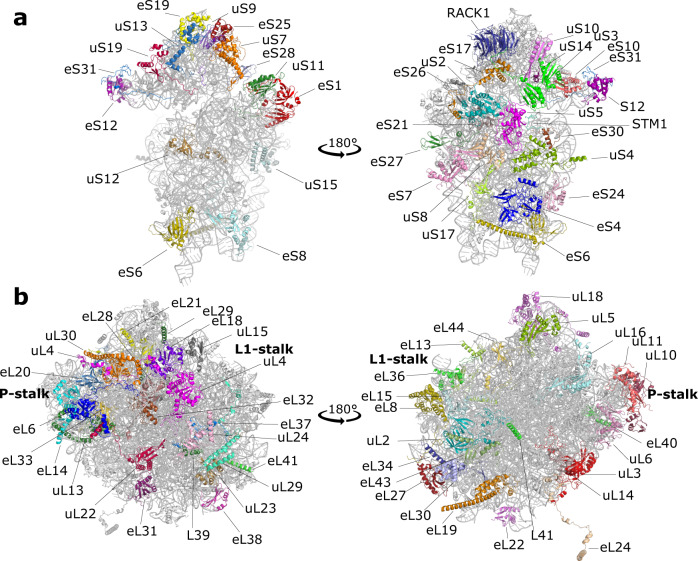
Overall architecture of the *Ct*80S ribosome. **a** The 40S subunit viewed from the intersubunit interface (left) and in the crown view (right) with ribosomal proteins indicated. **b** Same presentation for the 60S subunit from the backside (left) and in the crown view (right). The L1-stalk is missing and the P-stalk only partially built.

In order to obtain suitable samples of *Ct*80S ribosomes for high-resolution single-particle cryo-EM analyses, we adapted well established protocols for the purification of mesophilic and mammalian 80S monosomes (Supplementary Fig. 1)^32,33^. Cryo-EM reconstructions have been refined to an average resolution of 2.9 Å for the idle POST and 3.0 Å for the pe/E (TI)-POST state (Supplementary Figs. 2 and 3, Supplementary Table 1). Both states are devoid of mRNA and only the (TI)-POST state contains an undefined tRNA bound in the pe/E hybrid conformation. The resolution enabled us to build a high-precision model for a cytoplasmic 80S ribosome and to re-evaluate mechanisms of protein translation. The general architecture of the *C. thermophilum* 80S ribosome (Fig. 1) is similar to *T. lanuginosus* and *S. cerevisiae*. It contains 80 core RPs that are present in all eukaryotic cytoplasmic 80S ribosomes. *C. thermophilum* specific RPs were not found, based on complete density interpretation. On the contrary, some of the identified and built RPs were not annotated in the online resource for the *C. thermophilum* genome^34^ and a few proteins did not match the sequence deposited in the database (Supplementary Table 2). Due to its intrinsic flexibility, the L1-stalk was not built in either of the two structures.

**Fig. 2:**
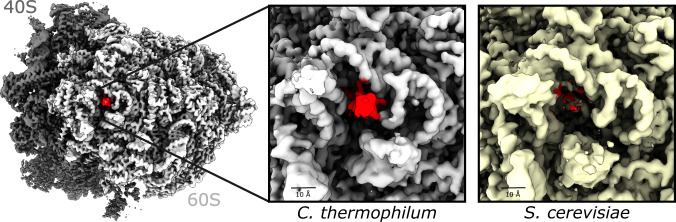
The second copy of eL41 at the periphery of the 60S subunit. Cryo-EM maps of 80S ribosomes from *C. thermophilum* and *S. cerevisiae*^1^. The second copy of eL41 (red) in *C. thermophilum* is tightly surrounded by the 26S rRNA, while the absence of eL41 in *Sc*80S ribosome leaves a deep protrusion in the ribosomal surface (void with red spots).

The presence of *Ct*80S RPs was validated by mass spectrometric analysis using intensity-based absolute quantification (iBAQ)^35^ (Supplementary Fig. 4 and Supplementary Table 3). Altogether, 240 proteins were identified. A population of 69 highly abundant proteins is separated from the bulk of 150 proteins, which are at least 10 times less abundant, whereas 21 proteins show intermediate abundance (Supplementary Fig. 4). The 69 highly abundant proteins are ribosomal subunits including RACK1 (1:1 stoichiometry). Non-ribosomal proteins present at sub-stoichiometric amounts to the ribosome are Stm1 (18%) and eEF2 (8%, corresponding to percentage of cryo-EM particles for (TI)-POST state). Some of the RPs have a low iBAQ value, because of their wrong annotation as longer versions (e.g., eL19 with 2898 aa)^34^. As intensity-based absolute quantification is normalized to the size of the protein, the iBAQ values for these proteins change to RP-average upon database correction (Supplementary Table 3). Generally, RPs are present as single copies (except for P-stalk proteins); however, in *Ct*80S we detect a second copy of the small α-helical protein eL41 (not detected by MS due to a lack of suitable tryptic peptides). eL41 is present in all eukaryotes and localizes at the interface between the 60S and 40S subunits. In *Ct*80S a second copy of eL41 is located at the periphery of 60S subunit, where it fills a hollow between surrounding rRNA (ES19 and ES26 in 26S, and ES3 in 5.8S). The second copy has so far not been found in other eukaryotic ribosomes and might be unique for *C. thermophilum* (see below; Fig. 2 and Supplementary Fig. 5).

Even though the *Ct*80S ribosome is highly homologous to *S. cerevisiae* 80S (*Sc*80S), it also shares similarities to the human ribosome, especially concerning RP tails. For example, while *S. cerevisiae* eS26 has a short C-terminus, the *C. thermophilum* protein has an extended C-terminal tail (even longer than in *H. sapiens* 80S (*Hs*80S)) bridging over the mRNA exit channel (Supplementary Fig. 6a). Another example is eS6, which in *S. cerevisiae* has a short α-helical C-terminus. In *C. thermophilum* RP eS6 extends towards expansion segment 6 (ES6), and in human ribosomes it contacts ES6^2^ (Supplementary Fig. 6b). In *Ct*80S, RPs eL6 and eL28 interact with ES7 thus stabilizing and fixing its position on the ribosomal surface. This interaction has also been observed in the *Hs*80S, but is not present in the *Sc*80S ribosome (which lacks eL28) (Supplementary Fig. 6c).

In order to analyze the stability of *C. thermophilum* ribosomes, we compared them with ribosomes from mesophilic *S. cerevisiae (Sc)* using nanoDSF. The melting temperature of *Ct*80S is 22 °C higher than for *Sc*80S (*T*_m_ of 62.5 °C and 40.5 °C, respectively) (Supplementary Fig. 7). While we exploit this increased stability of *Ct*80S primarily as a tool to obtain high-resolution structures, it implies significant adaptations, which are, however, difficult to dissect in a quantitative manner. Overall, thermophilic RP adaptations concern amino acid composition (bulky IVYWREL amino acids +5%^36^, arginines +11%, and prolines +13%^37^) as found in bacteria and archaea, and as also validated for *C. thermophilum*^27^. Increased bulkiness also confers to protein length, and *Ct*80S RPs often have terminal extensions compared with yeast. Whether thermophily of *Ct*80S also relates to the generally higher abundance of some RPs^38^ or their higher affinity to rRNA^39^ as observed for thermophilic bacteria is not known so far. A key factor that usually contributes to the increased stability is the G + C content of the rRNA^40^. *C. thermophilum* 18S and 26S rRNA have a 4.5% higher G + C content than *S. cerevisiae*, and this trend also shows for the 5S and 5.8S rRNA, however, less pronounced (Supplementary Table 4). However, the G + C content is similarly increased in the mesophilic fungus *Chaetomium globosum* and thus might also be a phylogenetic attribute of certain molds. We also observe an increase of repeats of three or more nucleotides with the same nucleobase (G and C homoiterons), which are known to support RNA secondary structure and stability of association with RPs^41^ (Supplementary Fig. 8). Such repeats are particularly common in ESs, where they partake a crucial role in reducing the flexibility of the peripheral rRNA.

The general analysis of the *C. thermophilum* rRNA revealed a total of 1794 nucleotides for 18S rRNA and 3338 nucleotides for 26S rRNA, numbers similar to *S. cerevisiae* and sharing 89% and 85% sequence identity, respectively. The rRNA structures in *Ct*80S are analogous to *Sc*80S, with the most obvious differences in the ESs (Supplementary Figs. 9–11). For 18S rRNA, especially *C. thermophilum* ES7 is much shorter than human ES7, and even slightly shorter and more compact than the yeast one. For 26S rRNA, segments H15, H30, ES19, and ES27 are shorter than their counterparts of the *Sc*26S. G + C repeats are very common in ESs, with e.g. ES27 having four additional homoiterons alone. Also, the *Ct*18S ES6A and ES6B, and ES12 adopt different conformations than their yeast counterparts and contain more G + C homoiterons (Supplementary Fig. 9). Taken together, the general criteria described for thermostability are met by the *Ct*80S RPs and rRNAs, and the high stability of *Ct*80S allowed us to determine high-resolution structures in two rotational states.

### Nascent chains and exit tunnel constrictions

**Fig. 3:**
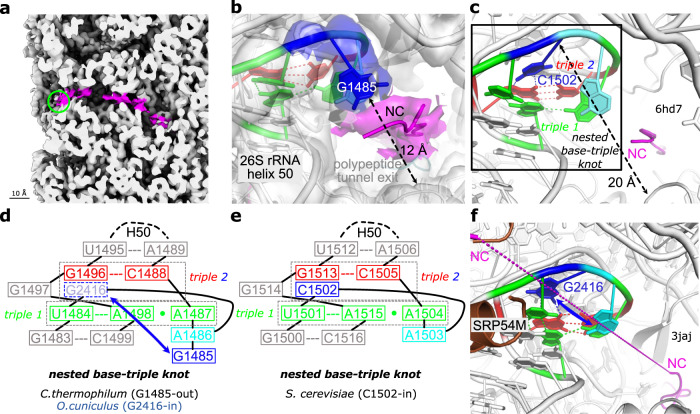
*Ct*80S ribosomes retain nascent chains. **a** Cryo-EM map for the (TI)-POST state of *Ct*80S with NC density shown in magenta. The narrowing at the ribosomal tunnel exit is highlighted by a green circle. **b** Zoom into the *C. thermophilum* 80S tunnel exit region covered by cryo-EM-density (3.5 σ, 1.5 σ for NC) with highlighted nucleotides of 28S rRNA H50. The tunnel diameter and relevant hydrogen-bonds (colored dashed lines) are indicated. **c** Comparison of the tunnel exit to *S. cerevisiae*^42^. Helix H50 forms a unique ‘nested base-triple (pseudo-)knot’. With the flipped-in C1502 (corresponding to G1485) two base-triples are formed. Same color code as in panel **b**. **d**, **e** Schemes of the nested base-triple knot with two intertwined triples and an exposed base stack as platform for the bulged-out nucleotide, as shown for *C. thermophilum* (**d**) and *S. cerevisiae* (**e**). Base-flipping is indicated for the respective *O. cuniculus* G2416 nucleotide (light blue, dashed outline) as revisited here for the mammalian SRP-ribosome complex^4^. **f** Mammalian SRP-ribosome complex^4^. The close-by SRP54M domain recognizes the N-terminal signal (upper left corner) of the NC in the tunnel (parts connected by dotted line). The guanine base is built flipped-in, but according EM-density is at least partially bulged-out (indicated by double-arrow, see also Supplementary Fig. 20).

The cryo-EM reconstructions show that the *C. thermophilum* ribosomes contain density throughout the exit tunnel in both the idle and pe/E (TI)-POST states, which can be explained satisfactorily only as mixture of nascent chains (NCs) (Fig. 3a). This came as a surprise, as puromycin (a protein synthesis inhibitor) treatment during ribosome preparation typically results in premature NC termination during translation and the growing peptides are released as peptidyl-puromycin^42^. However, puromycin treatment of *Ct*80S did not result in significant NC release and our iBAQ analyses show no additional stress factors apart from Stm1. Following a purification protocol adapted for *S. cerevisiae* ribosomes, we initially performed puromycin treatment of *Ct*80S at 30 °C. As *C. thermophilum* grows at 50–55 °C, we tested whether NC stalling can be overcome at higher temperature and performed puromycin incubation of *Ct*80S at 50 °C. Subsequent cryo-EM structure determination again resulted in the idle and pe/E (TI)-POST states that both still contained NCs in the exit tunnel revealing that puromycin treatment at higher temperature also does not release NCs from *Ct*80S. In contrast, human ribosomes analogously prepared with the same puromycin batch did not retain any NCs as judged by cryo-EM.

In order to see whether there is a structural explanation for the presence of NCs in *Ct*80S, we carefully inspected the polypeptide exit tunnel. The observed density for the NCs results from a mixture of different peptides present in the exit tunnel and was modelled as a poly-alanine chain of 25 amino acids. In both rotated states, we were able to trace the NC from the beginning of the empty peptidyl transferase center (PTC) up to the very tunnel exit. Closer inspection shows the two established constrictions (Supplementary Fig. 12a). The first one is created by uL4 and uL22 loops, localizes at the NC residues 10–12, and is conserved in all domains of life^43,44^. The second one is created by the uL4 protein, localizes at NC residue 16, and is specific for eukaryotic ribosomes^44^. However, apart from these two conserved constrictions, we observe a third constriction at the very end of the *Ct*80S exit tunnel that has previously not been described. Here, guanosine G1485 of 26S rRNA H50 is bulged-out towards the tunnel thus narrowing the passageway to only about 12 Å (Fig. 3b and Supplementary Fig. 12b). G1485 forms the largest direct contact with the NC. For comparison, in *Sc*80S (without NC) the corresponding cytosine (C1502) is not bulged-out and hidden in the 26S rRNA^45^, which results in a much wider diameter of the exit tunnel of about 20 Å (Fig. 3c and Supplementary Fig. 12c). In a cryo-EM structure of the yeast ribosome/NatA (pull-out with a mixture of NCs)^45^, C1502 seems to remain hidden in the rRNA, however, also with no electron density of the NC visible next to this cytosine. This suggests that in *Ct*80S the NCs might be stabilized in the exit tunnel by the additional interaction with the bulged-out G1485.

Guanosine G1485 is not only present in *C. thermophilum*, but also in the 28S rRNA of mammals and other higher eukaryotes. It is the central element of an unusual minimal nested RNA pseudoknot, which we describe as a ‘nested base-triple knot’ with two intertwined triples and an exposed base stack forming a platform for the bulged-out nucleotide (Fig. 3d). This rRNA pseudoknot has so far not been annotated although it is present in ribosomes of all eukaryotes (e.g. *H. sapiens*, *S. cerevisiae*; Fig. 3e), prokaryotes (e.g. *E. coli* or *Thermus thermophilus*^46^) and archaea (e.g. *Haloarcula marismortui*^47^) structurally characterized so far, and based on sequence comparisons it seems universally conserved (Supplementary Fig. 13). The *Ct*80S structures of this study are to our knowledge the first reported examples with a bulged-out nucleotide originating from one of the base-triples (base-triple 2). In order to analyze this further, we carefully revisited mammalian 80S cryo-EM structures obtained with and without NCs. In a recent 3.0 Å 80S structure with ribosomes stalled on the NC of the ribosome-arresting XBP1u protein^48^, the EM-density indicates at least partial flipping of the base into the tunnel although this feature (and the adjacent NC) was not built. Similarly, in a stalled mammalian RNC in complex with the signal recognition particle (SRP)^4^ (Fig. 3f), the EM-density also indicates that the nucleotide is partially bulged-out although it was not built. Finally, in the high-resolution cryo-EM structure of the idle human 80S ribosome^49^, the careful re-evaluation of the cryo-EM-density indicates nucleotide dynamics also in the absence of a NC. Taken together, these observations show that this base-flipping into the exit tunnel can occur independent of a NC and is a more general feature that so far has escaped attention.

### Two conformations of the ribosome-associated factor Stm1

**Fig. 4:**
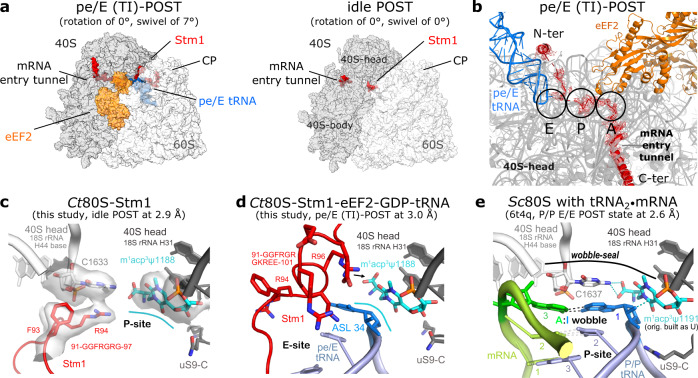
Ribosome-associated factor Stm1 and the m^1^acp^3^Ψ hypermodification. **a** Surface representation of the *Ct*80S in both rotational states with bound Stm1 (red), eEF2 (orange), and pe/E tRNA (blue). **b** Zoom on the pathway of Stm1 following the P- and A-sites, and the mRNA entry tunnel. A-, P-, and E-sites are marked with circles. **c** In the idle POST state Stm1 occupies the mRNA position in the P-site. The strictly conserved m^1^acp^3^Ψ hypermodification in eukaryotic 18S rRNA (*Ct*U1188) lines the P-site (cyan line). Cryo-EM map is shown for central features (2 σ). **d** In the rotated (TI)-POST state of *Ct*80S, Stm1 conformation is changed and m^1^acp^3^Ψ1188 forms an interaction with the pe/E-site tRNA-ASL in the E-site. **e** In presence of the codon-anticodon^2^, the modified nucleotide (not built originally) forms a lid described here as *wobble-seal* together with a conserved cytosine stacking on the wobble.

Both of our *Ct*80S structures contain the ribosome inhibitory protein Stm1 (Suppressor of target of Myb protein 1). Stm1 and its analogues have so far only been described in context of rotated ribosomal states^1,2,50^. Interestingly, we observe Stm1 not only in the rotated pe/E (TI)-POST state with bound eEF2-GDP but also in the back-rotated idle POST state, although in a markedly different conformation (Fig. 4a, b). We could not assign the entire polypeptide of Stm1, but for the (TI)-POST state we were able to build residues 26–49, 60–80, and 89–154. Here, Stm1 adopts an extended toothpick-like structure starting from the central protuberance (CP) of the 60S up to 40S body/head interface, following the mRNA track backwards and passing through the P- and A-sites (here occupying the codon-anticodon space), and the mRNA entry tunnel. In the CP, Stm1 wraps around the 5S rRNA, H84 of 26S rRNA, uL5, and eL42 (Supplementary Fig. 14a). Stm1 then passes the intersubunit bridge B2a and thus stabilizes the rotated state as observed earlier^50^. Of note, the intersubunit bridges in *Ct*80S are similar as reported for other eukaryotic ribosomes^1,3^ (Supplementary Table 5). Importantly, Stm1 not only occupies important functional sites of the ribosome, but also inserts an extended α-helix (residues 127–154) in the 40S body/head interface next to the mRNA entry tunnel, thus interfering with back-swiveling of the 40S head (Supplementary Fig. 14b). The extended α-helix forms two salt bridges to conserved arginines of uS3 that are known to be important for pre-initiation complex stabilization^31^. The mRNA entry tunnel is entirely blocked by Stm1 (111-126), located between uS3 and uS5. Multiple contacts with the 40S include uS3, eS5, eS10, eS12, uS13, uS19, a tight interaction with 18S rRNA H18, and an interaction with eEF2 (Supplementary Fig. 14c). The multitude of Stm1 interactions seems important for preventing subunit dissociation under nutrient-deficient conditions as shown in yeast^30^.

In contrast, in the non-rotated (and non-swiveled) idle POST state we could localize only two short Stm1 fragments bound to the 40S (residues 87–97 and 127–147), indicating higher flexibility in this state (Fig. 4a). Here, the extended α-helix (residues 127–154) is dissolved and residues are folded onto the 40S body instead, and the swiveling-block is released (Supplementary Fig. 14d, e). Electron density is not visible at the 60S subunit or in the mRNA entry tunnel and the A-site, and Stm1 binding to the P-site is also altered with Stm1 folding into an open hairpin structure.

The A- and P-sites in both *Ct*80S structures are devoid of mRNA and tRNA (pe/E state frees major parts of P-site) and Stm1 fills the mRNA-void with an extremely flexible and surprisingly positively charged sequence fingerprint (89-RRGGFRGRGKREE-101). In the idle POST state, Stm1 Arg94 takes the wobble position of the mRNA within the P-site and is involved in π-cation stacking with an invariant cytosine (*Ct*C1633) from H44 of 18S rRNA (Fig. 4c). The swiveling of the 40S head in the (TI)-POST state shifts Arg94 above the wobble position allowing the Stm1 region around the 94-RGR motif to contact the anticodon of the pe/E tRNA (Fig. 4d). Comparison with a yeast 80S structure containing the mRNA (Fig. 4e) shows that Stm1 mainly occupies the mRNA binding region. With its positively charged residues taking the space of the mRNA bases it can be regarded here as an mRNA placeholder. Having observed this particular binding mode of Stm1, we performed a sequence comparison with the corresponding proteins in yeast and humans (Supplementary Fig. 15). Sequence conservation is rather local, like e.g., a WG-motif (*Ct*W123) binding in the mRNA entry tunnel, or comprises extended RG-repeats occupying the A- and P-sites as found in this study. These repeats are also found in the C-termini, which are not resolved in any cryo-EM structure.

### Protein and rRNA modifications

**Fig. 5:**
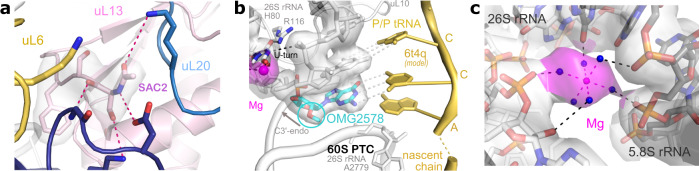
*Ct*80S modifications and ligands. **a** N-terminal acetylation of Ser2 of uL13 (SAC2) is shown in its cryo-EM-density and with close interactors (highlighted by color and with dashed connector lines). **b** Conserved 2’-O methylation in the PTC. The high resolution allows for detailed modelling of sugar methylations and conformations (OMG2578), and of magnesium coordination. The peptidyl-tRNA is modeled from a superposed high-resolution *Sc*80S structure^2^. **c** Typical example for a magnesium ion (magenta) with its hydration shell tethering two rRNA helices of 5.8S and 26S rRNA, respectively. Interactions are indicated by dashed lines. All cryo-EM maps are contoured at a 2.5 σ level.

In our structure of the idle *Ct*80S ribosome at 2.9 Å extra density for putative chemical modifications is present as described in detail for other eukaryotes^3,51,52^. However, modifications have not yet been analyzed and validated (e.g., by mass spectrometry) for *C. thermophilum* ribosomes. Therefore, we focus here on two strictly-maintained rRNA modifications and two examples for protein modifications, an N-terminal RP acetylation and the diphthamide (Dph) modification of eEF2 (see below). N-terminal acetylation is a widespread protein modification among eukaryotes and alters lifespan, folding characteristics and binding properties of the acetylated proteins^53^. N-terminal acetylation of RPs was also shown to influence translational fidelity in yeast^54^. In our MS analysis, 31 out of the 70 most abundant RPs were found to be modified and most often when the methionine is cleaved-off. Interestingly, these RPs are most of the time detected in similar amounts with or without modification (Supplementary Table 3). As an example of how N-terminal acetylation stabilizes protein-protein interactions in the ribosome, uL13 Ser2-Ac of the 60S subunit is shown (Fig. 5a).

Most of the rRNA modifications are known to cluster around the central functional sites as the PTC, A- and P-sites, the DC, or intersubunit bridges^52^. In eukaryotes, the most abundant RNA modifications are 2’-O methylation of the ribose and the isomerization of uridine to pseudouridine (Ψ)^52^. For example, in the PTC the penultimate cytosine of the peptidyl-tRNA forms a Watson-Crick base pair with a conserved guanine that always carries a ribose methylation (OMG2578 in *Ct*26S rRNA). Despite the absence of the P-site tRNA, this methylation is clearly identified in our idle *Ct*80S structure, highlighting the level of detail resolved in high-resolution cryo-EM reconstructions (Fig. 5b). The modification is found at the tip of the short H80 stem-loop with the 2’-O-Me moiety in plane with the nucleotide base, which has been described to be important for extending base-stacking^49^. The quality of the density allows us to confirm the sugar pucker as the 3’-endo conformation as observed for non-flipped rRNA bases^49^. The same region also highlights the importance of magnesium ions for ribosome integrity, and overall, more than 550 ions could be localized with many of them tethering rRNA helices (Fig. 5c). The H80 closing uridine (U2576) forms a U-turn and is ligated to magnesium as a mediator to close phosphates and bound to an uL10 arginine side-chain (R116) at the heart of the PTC, respectively.

### A function for the unique m^1^acp^3^Ψ hypermodification

An important and conserved rRNA modification in eukaryotic ribosomes is the 1-methyl-3-α-amino-α-carboxyl-propyl pseudouridine (m^1^acp^3^Ψ) hypermodification in 18S rRNA of the 40S head^55^. In our idle *Ct*80S structure, m^1^acp^3^Ψ (U1188 in *Ct*18S, U1191 in *Sc*18S, and U1248 in *Hs*18S rRNA) lines the P-site at the tRNA wobble position (Fig. 4c) as also found in the idle human 80S ribosome^49^. The mRNA codon wobble position is filled by Stm1 as described above. The comparison with a yeast 80S cryo-EM structure, including mRNA and P-site tRNA stalled on an obligate A:I wobble base pair in the POST state (modification not built, but defined in the same orientation)^5^, allows to derive a specific role for this modification. It forms a ‘*wobble-seal*’ with intimate contacts to the nucleotide in position 1 of the anticodon (Fig. 4e). In detail, while the α-amino group hydrogen-bonds to the inosine phosphate as seen in the A:I wobble, the α-carboxyl binds to the exocyclic N2 of an invariant cytosine of 18S rRNA (*Ct*C1633, *Sc*C1637, *Hs*C1701; at the base of H44) that stacks on top of the wobble base pair. Moreover, the plane of the modified base, extended by the 1-methyl group, stacks upon the inosine-ribose, as typical for carbohydrate-binding to hydrophobic side chains. These interactions indicate that the hypermodification is essential to close the seal over the wobble. Prokaryotes do not have this modification, however, the exchange of the uracil for the larger and (m^2^)-modified guanine (essential for bacterial fitness^56^) perfectly matches the hypermodification as defined for the *E. coli* system^57^ (Supplementary Fig. 16). Here, as also in yeast^5^, a conserved arginine at the C-terminus of uS9 aids tRNA binding by stabilizing the anticodon from the side. This arginine is exchanged in *C. thermophilum* to a lysine that is turned away (Fig. 4c).

Interestingly, despite the (TI)-POST structure presenting an off-pathway intermediate, it corresponds to a late intermediate of translocation according to its rotational state^58^. During rotation between our two observed states, m^1^acp^3^Ψ1188 follows the swiveling motion as part of the 40S head; however, it still binds to the ASL of the translocating pe/E tRNA the same way as it would as part of the *wobble-seal* with a PRE P/P tRNA as described above (movement of 12.5 Å; Fig. 4d and Supplementary Fig. 17). Due to the tight interaction of this large rRNA modification grabbing around the whole nucleotide, and the importance of the ASL for tRNA binding^10^, it seems likely that the tRNA rotates back and forth during ribosomal Brownian-ratcheting together with m^1^acp^3^Ψ1188 and staying in direct contact throughout.

### The eEF2-GTPase switch cycle and ribosomal rotation

**Fig. 6:**
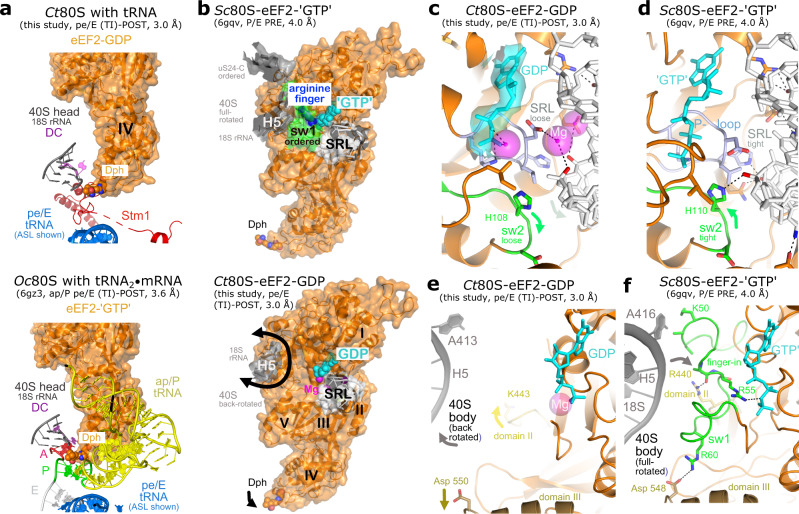
eEF2 Dph modification and GTPase switch cycle. **a** Top: (TI)-POST state of *Ct*80S with eEF2-GDP shows the position of the conserved diphthamide (Dph) histidine modification at the apex of eEF2 domain IV. The monitoring adenines of the DC are marked in magenta. Bottom: The (TI)-POST state with tRNA_2_•mRNA module^56^ (although still with non-hydrolyzable eEF2-‘GTP’). Dph is in same post-conformation as in our (TI)-POST state. Codons and associated tRNAs are color-coded. **b** The rotated ribosome acts as GAP for GTP hydrolysis in eEF2. Top: The full-rotated ribosome^2^ (40S body helix H5) pushes on the ordered switch 1 region (sw1) of eEF2 and places an intrinsic arginine finger. Bottom: Upon back-rotation (indicated by arrows), as observed in the (TI)-POST state of *Ct*80S with eEF2-GDP-Mg^2+^, sw1 becomes disordered. **c**, **d** Switch 2 (sw2, green) conformations during the eEF2 switch cycle. In context of the PRE ribosome, the SRL is in tight contact with sw2 and the P-loop (light blue) of eEF2-‘GTP’^2^. Upon GTP hydrolysis and as detailed for (TI)-POST *Ct*80S, the P-loop and sw2 relax and contacts are loose. Magnesium ions (magenta spheres) mediate SRL-eEF2 contacts. GDP is shown with its cryo-EM map (3 σ). **e**, **f** Switch 1 conformations during the eEF2 switch cycle. In the full-rotated state, the arginine finger within sw1 is placed on top of the scissile bond (finger-in) and the ribosome acts as GAP^2^ (not described there). Upon back-rotation of the 40S body (indicated by gray arrow), as seen in the (TI)-POST state of *Ct*80S, sw1 becomes disordered and eEF2 relaxes in a relay system (distant domains II and III).

The elongation factor eEF2 undergoes large conformational changes upon ribosome binding and rotation^18^. Our late pe/E tRNA (TI)-POST ribosome structure allows the assignment of eEF2-GDP-Mg^2+^ that corresponds to the GTP post-hydrolysis conformation just before eEF2 leaves the full-translocated ribosome. In this state, the Dph modification of a histidine (*Ct*H701) at the apex of eEF2 domain IV is located deeply inserted into the ribosomal A-site and is surrounded by the Stm1 protein (Fig. 6a and Supplementary Fig. 14c). Overall eEF2-ribosome interactions are detailed in Supplementary Fig. 18. Although the Stm1-bound structure is an off-pathway intermediate and contacts are weak, the Dph modification is found in the same position as observed in structures of the mammalian ribosome in different (TI)-POST states including a complete tRNA_2_•mRNA module^10,18^ (Fig. 6a). Here, Dph was found in tight contact with the minor groove of the P-site codon-anticodon, whereas in the PRE state^24^, it has been found moved outwards from the A-site in close contact to the so-called ‘monitoring adenines’ of the DC. In absence of a tRNA_2_•mRNA module, our late pe/E tRNA (TI)-POST state bound to eEF2-GDP reveals the monitoring adenines of the DC hidden in H44 of the 40S head (Fig. 6a).

Efficient translocation of the tRNA_2_•mRNA module of the processive ribosome relies on eEF2-GTP hydrolysis. Distant from Dph, the active site of the eEF2-GTPase is located in domain I, which is hooked on the SRL^24,59^ (Fig. 6b). Our pe/E tRNA (TI)-POST structure with eEF2-GDP-Mg^2+^ now provides insights into a late (TI)-state after GTP hydrolysis (Fig. 6c). GTP hydrolysis relies on two flexible switch regions (1 and 2) of the GTPase. The switch 2 region, harboring the catalytic histidine (*Ct*H108) is ordered and two magnesium ions are found to bridge between negatively charged side chains and the phosphoribose backbone of the SRL. Comparing unbound elongation factors (EF-G or eEF2) with ribosome-bound ones^24,60^ shows how the SRL generally pushes on the GTPase switch 2 region (Fig. 6c, d) and thereby activates the catalytic histidine, which flips into the active site to position the catalytic water molecule (not modelled in other cryo-EM structures). GTP hydrolysis, and probably subsequent phosphate release, as shown very recently by detailed time-resolved cryo-EM studies for the bacterial system^61,62^, impacts on eEF2-ribosome interactions by modulating and loosening contact sites, as observed in our eEF2-GDP structure between the P-loop and the SRL.

GTP hydrolysis also requires ordering of the switch 1 region to allow completion of the active site usually by an arginine finger, which is often supplied for GTPase activation either *in cis* or in trans by a GAP (GTPase-activating protein)^63^. In our (TI)-POST structure the whole switch 1 effector loop (residues 42–67) of eEF2 is disordered (Fig. 6b, e) indicating major rearrangements along the GTPase switch cycle. A high-resolution X-ray structure of eEF2-GTP is lacking, however, also in a bacterial EF-G-GMPPNP structure^60^ switch 1 is disordered and an arginine finger was not defined. Mutational studies on switch 1 arginines of *E. coli* EF-G only showed moderate inhibition and thus an arginine finger hypothesis was discarded^64^. Interestingly, in an 80S-eEF2-GMPPCP structure in context of the full-rotated PRE ribosomal state^24^ as well as in previous low-resolution structures^18,19^, switch 1 adopts a defined conformation and establishes a contact to the 40S body (*Sc*A416 of H5 in 18S rRNA) (Fig. 6b). Although not described, in this full-rotated structure an inbuilt arginine finger (*Sc*Arg55 of eEF2, *Ct*Arg55) is indeed placed on top of the scissile bond (Fig. 6b, f). Interestingly, a corresponding arginine seems to be absent in bacteria and archaea (Supplementary Fig. 19). Of note, eukaryotic (and archaeal) switch 1 loops have a four-residue deletion inducing a conformational change, which makes a direct structural comparison difficult.

Finally, the ordering of the eukaryotic switch 1 region is transmitted to eEF2 domains II and III by a relay system. As deduced from yeast eEF2^24^ (Fig. 6f), a positively charged residue (*Sc*Arg440) on the tip of an extended hairpin of domain II is fixed in a pocket below the arginine finger. Similarly, domain III is held via at least one salt bridge (*Sc*Arg60/Asp548). However, in our (TI)-POST eEF2-GDP structure, the conformational changes induced upon GTP hydrolysis and phosphate release result not only in a disordered switch 1, they also abrogate the contacts with domains II and III that transmit the hydrolysis event to domain IV (Fig. 6e). Overall, eEF2-GDP is now ready to leave the ribosome.

## Discussion

Understanding the molecular mechanisms of the ribosome has for decades been a central theme in molecular cell biology. Here, we provide structural data for the thermophilic fungus *Chaetomium thermophilum* and deduce functional implications for the eukaryotic 80S ribosome integrating the vast database of ribosome research. The structural analyses are complemented by quantitative mass spectrometry (iBAQ). The iBAQ data nicely mirror the structural findings by giving an inventory for the ribosome and its associated factors, and reveal important information on the N-terminal acetylation status of the RPs. The high-resolution structures provide a blueprint for a thermophilic 80S ribosome and give some insights into thermal adaptations both on protein and RNA levels. Overall, RPs contain more bulky side chains and elongated tails; and in a unique case an eL41 duplicate fills an rRNA packing void. The rRNA shows an increased GC content with respective homoiterons as known for bacterial thermophiles^37^, and the ESs are shorter than in mesophiles. Interestingly, both substrate exit tunnels of the ribosome are constricted. The polypeptide exit tunnel is narrowed at the very end by the 26S rRNA and the mRNA exit tunnel by the eS26 C-terminal tail (Supplementary Fig. 20). However, while the mRNA is not present, the NC appears trapped in the exit tunnel. Whether and how chemical modifications and ligand binding (e.g., metals) contribute to thermostability still needs to be carefully analyzed in subsequent studies.

The increased stability of the thermophilic ribosome directly impacts on the cryo-EM reconstructions reflected by structural features that were previously not resolved. An intriguing example is the identification of a universally conserved rRNA structural element in the polypeptide exit tunnel, which induces a significant constriction of the exit tunnel by a bulged-out nucleotide that likely prevents NC release despite puromycin treatment. This adaptable constriction seems to be a more general feature of eukaryotic ribosomes. While it is present in mesophilic 80S ribosome structures to a lower extent (so far undetected), the equilibrium of base-flipping is shifted in *Ct*80S. Revisiting a structure of mammalian RNCs in complex with the co-translational targeting SRP complex^4^, we find evidence for a partially flipped guanosine (G2416 in *Oc*28S rRNA) only 10 Å away from the SRP54M domain and about 25 Å from the signal peptide present in the structure (Fig. 3f and Supplementary Fig. 21). Therefore, bulging-out of this base might directly serve in NC handover to downstream factors. Already available cryo-EM reconstructions of mammalian RNCs in complex with ribosome-arresting NCs^5^ provide further evidence for such function.

Our structures represent snapshots of two rotational states along the eukaryotic ribosomal translocation cycle, although mRNA is missing: a late (TI)-POST state with pe/E tRNA and eEF2-GDP and the idle POST state. Both structures contain the ribosomal recycling factor Stm1, which acts as protective and stabilizing factor for hibernating ribosomes upon cellular stress conditions^30,31^. Stm1 stabilizes the rotated state by spiking through both subunits and occupying important functional sites following exactly the mRNA trace, as observed in our (TI)-POST structure. In this state, Stm1 is known to inhibit ribosomal subunit splitting by the Dom34 recycling system^50^. However, we find Stm1 also present in the non-rotated idle POST state, where it adopts a significantly different conformation leaving the A-site and mRNA entry tunnels empty. Here, these sites could be readily occupied by the Dom34 ribosomal subunit splitting protein (Supplementary Fig. 22) in the same manner as observed in yeast^50^. Thus, we reason that only in the rotated state Stm1 protects from subunit splitting. Further, the Stm1 structure at least partially resembles the binding mode of the alternative ribosomal rescue factor ArfB^65^. ArfB rescues bacterial and mitochondrial ribosomes on stalled nonstop mRNAs by releasing NCs from the P-site tRNA. The C-terminal parts of both proteins pass via the A-site and are threaded through the entire mRNA entry channel making mRNA binding impossible (Supplementary Fig. 23). However, while Stm1 follows the mRNA path, the folded N-terminal hydrolase domain of ArfB mimics a release factor by inserting a GGQ motif into the PTC^65^.

**Fig. 7:**
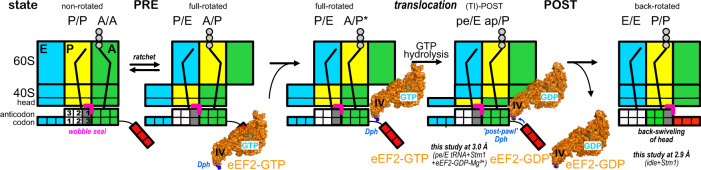
Scheme of eEF2-catalyzed ribosome translocation. From left to right: Schematic for eukaryotic ribosomal states along a translocation cycle starting with the PRE state after peptide-bond formation. The m^1^acp^3^Ψ hypermodification (magenta bracket) within the 40S head, on top of position one of the anticodon in the wobble, helps as part of the *wobble-seal* in coupling ribosomal rotation to tRNA_2_•mRNA module movements between the classical A/A P/P and the A/P P/E hybrid states. Dph (highlighted in blue) within domain IV of eEF2-GTP acts as a *doorstop* (*pawl)* in the A-site and the full-rotated state is stabilized. The full-rotated ribosome acts as GAP inducing GTP hydrolysis in eEF2, marking the transition to the translocated POST state. Back-rotation of the 40S head is uncoupled from the tRNA_2_•mRNA module and Dph acts as ‘*post-pawl’* in contact with the P-site. eEF2-GDP binding is weakened due to the internal relay and SRL-contact loosening and upon back-swiveling of the 40S head, eEF2-GDP is released. Translocation is complete in the P/P E/E state.

The high resolution of both *Ct*80S structures permits to build protein and RNA modifications with atomic precision and, in context with previous ribosomal data, provides new insights into the translocation process (Fig. 7). We observe the unique m^1^acp^3^Ψ hypermodification within 18S rRNA in the 40S head in atomic detail and describe it as an essential component of a codon-anticodon *wobble-seal* in the P-site. Its central importance is underlined by the loss of this modification (or hypo-modification) being implicated in about 50% of all rectal colon cancers^55^. Due to its size and presence of additional functional groups, it ensures a tight association with the anticodon position 1 that seems to be important for wobble stability and for efficient and in-frame translocation of the tRNA_2_•mRNA module from the P- to the E-site. Furthermore, comparing our and other (TI)-POST structures including the complete tRNA_2_•mRNA module^10^, allow for the assignment of the Dph conformation to a defined post-state (a Dph *post*-*pawl* position) upon GTP hydrolysis. The post-state conformation of domain IV corresponds to the time-resolved cryo-EM data obtained very recently for EF-G^62^, however, Dph is not present in bacteria.

Finally, we refine the GTPase switch cycle of eEF2. Although, the pe/E tRNA-bound state in complex with eEF2 is common for structures determined upon puromycin treatment of mammalian and yeast ribosomes^50,66^, the nucleotide load could not be assigned previously due to limited resolution and/or the mechanistic definition of the eEF2 switch cycle was not in the focus of these studies. Our *Ct*80S (TI)-POST state shows eEF2 in the GDP-Mg^2+^ bound state in relatively loose contact with the SRL (mediated by magnesium ions) and the switch 2 region within the GTPase domain I. As expected after GTP hydrolysis and phosphate release, the switch 1 region is disordered. Our structure is similar to 80S-eEF2-GDP-IRES structures from yeast stabilized by the antifungal sordarin^59^. This study defined 80S rotational dynamics of in total five eEF2-GDP-bound intermediates; however, the eEF2-GTPase switch cycle was not described. Further, revisiting and integrating previous structures with non-hydrolyzable eEF2-‘GTP’^10^ reveals an intrinsic arginine finger within switch 1 that is susceptible to the ribosomal rotation state. Only in the transient full-rotated PRE state bound to eEF2-GTP, the 18S rRNA within the 40S body pushes on switch 1 and places the arginine on top of the scissile bond. Thus, comparison of our ribosomal eEF2-GDP structure to the eEF2-‘GTP’ structures shows that the full-rotated ribosome acts as classical ‘GAP’ (GTPase-activating protein) for eEF2: the rotated 18S rRNA places the switch 1 arginine finger and the SRL the switch 2 catalytic residue. Thus, it is not eEF2-GTP that stabilizes the full 80S rotation, but the rotation in situ ‘stabilizes’ eEF2-GTP in the full-activated conformation. How bacterial EF-G is activated by the rotating ribosome is left unexplained here, but recent time-resolved cryo-EM data^61^ indicate a different mechanism of sw1 compaction without an arginine finger and including a SRL twisting that modulates sw2 in the transition state of GTP hydrolysis.

Of note, eEF2 is a member of the ancient TRAFAC (translation factor) superfamily of GTPases^67^ that co-evolved with the ribosome at the same time as the SRP GTPases (SRP54, SRα) regulating co-translational targeting. Most TRAFAC GTPases seem to be activated by RNA (this study and refs. ^68–70^) and thus open a window to an ancient eukaryotic RNA world. While the GTPase switch cycle is stimulated by ribosome binding, translocation per se is uncoupled from GTP hydrolysis and it is only eEF2 release that depends on it^23^. Upon back-rotation of the 40S subunit, phosphate release is communicated in a relay system via switch 1 to the adjacent domains II and III. Finally eEF2 relaxes, in agreement with the general ‘loaded-spring’ model^63^, as discussed also for EF-G^61^. The back-swiveling of the 40S head as last step of translocation is then able to release eEF2-GDP and to set the stage for a new round of polypeptide elongation.

## Methods

### Purification of *C. thermophilum* 80S ribosomes

The protocol for the isolation of *C. thermophilum* non-translating 80S (*Ct*80S) ribosomes was adapted from previously described methods^32,33^. *C. thermophilum* cells were grown in a rotary shaker at 55 °C for 3 d, harvested with a vacuum filter and immediately frozen in liquid nitrogen. Frozen mycelium cells were ground to fine powder by Cryo Mill (Retch) (5 min, frequency 30/s) and stored at −80 °C. The powdered mycelium was resuspended in 20 mM HEPES-KOH (pH 7.5), 500 mM potassium acetate, 5 mM magnesium acetate, 2 mM DTT, and 0.5 mM PMSF and vortexed until no clumps remained. Insoluble material was removed by centrifugation (48,254 × *g*, JA25-50 rotor (Beckman), 35 min). Ribosomes were pelleted through a high-salt sucrose cushion (20 mM HEPES-KOH (pH 7.5), 500 mM potassium acetate, 1.5 M sucrose, 5 mM magnesium acetate and 2 mM DTT) at 124,517 × *g* in a T-865 rotor (Thermo Scientific) for 18 h before they were resuspended in 20 mM HEPES-KOH (pH 7.5), 50 mM potassium acetate, 5 mM magnesium acetate, 2 mM DTT and 0.5 mM PMSF. The *Ct*80S ribosomes were then incubated with 1 mM neutralized puromycin solution and 1 mM GTP for 1 h at 30 °C (or 50 °C—in a high temperature control). Ribosomes were further purified in a 15–40% sucrose gradient (20 mM HEPES-KOH (pH 7.5), 150 mM potassium acetate, 5 mM magnesium acetate, 15–40% sucrose, 2 mM DTT and 0.5 mM PMSF) spun at 60,076 × *g* in a Surespin 630 rotor (Sorvall) for 15 h. Peak fractions containing *Ct*80S monosomes were pooled and precipitated by adding 7% (w/v) of PEG20K. After a 10 min centrifugation, the pellets were resuspended in 20 mM HEPES-KOH (pH 7.5), 50 mM potassium acetate, 5 mM magnesium acetate, 2 mM DTT and 0.5 mM PMSF, and used for cryo-EM grid preparation or stored at −80 °C.

### NanoDSF measurements

Melting temperatures (*T*_m_) of *C. thermophilum* and *S. cerevisiae* (purified as previously described^33^) ribosomes were measured using nano differential scanning fluorimetry (nanoDSF). Intrinsic tyrosine and tryptophan fluorescence at emission wavelengths of 330 nm and 350 nm were measured continuously applying a temperature gradient of 20–90 °C in the Prometheus NT.48 nanoDSF system. The *T*_m_ was calculated by the supplied software (NanoTemper Technologies GmbH).

### In-gel tryptic digestion, LC-MS/MS analysis, and database search

Samples were purified and separated by SDS-PAGE for 1 cm. Coomassie stained lanes were excised and processed as described previously^71^. In brief, samples were reduced with DTT, alkylated with iodoacetamide and digested with trypsin. Peptides were extracted from the gel pieces, concentrated in a SpeedVac vacuum centrifuge and dissolved with 15 μL 0.1% TFA. Nanoflow LC-MS2 analysis was performed with an Ultimate 3000 liquid chromatography system coupled to an Orbitrap Elite mass spectrometer (Thermo-Fischer, Bremen, Germany). Five microliters of sample were injected to a self-packed analytical column (75 μm × 200 mm; ReproSil Pur 120 C18-AQ; Dr Maisch GmbH) and eluted with a flow rate of 300 nL/min in an acetonitrile-gradient (3–40%). One survey scan (res: 60,000) was followed by 15 information dependent product ion scans in the ion trap. For quantification the MaxQuant software (1.6.12.0)^72^ was used. Database search was done against a *C. thermophilum* database downloaded from UniProt.org (Proteome ID 08066) and a custom database with additional sequences (Supplementary Table 3). In addition, the contaminants database included in the MaxQuant software was used. Trypsin was specified as enzyme. Carbamidomethyl was set as fixed modification of cysteine and oxidation (methionine), deamidation (asparagine, glutamine) and N-terminal acetylation as variable modifications. A false discovery rate of 1% was used on peptide and protein level. In addition, a minimum score of 40 was used for peptide identification. iBAQ calculation^35^ was enabled and LFQ normalization was disabled. Instead, LFQ values of the different experiments were normalized using the 70 proteins with the highest iBAQ values to exclude contaminants from normalization. iBAQ values based on these LFQ values were calculated using the number of iBAQ peptides provided by MaxQuant. To analyze modifications at the protein N-terminus, the proteome discoverer software 2.5 (Thermo Scientific) was used with the same settings regarding false discovery rate and peptide modifications. In addition to significant XCorr values, fragment spectra of modified protein N-terminal peptides were manually inspected. When several modifications were detected at the same protein N-terminus, intensity-based comparison was based on spectral counting.

### Cryo-electron microscopy grid preparation and data collection

Three microliters of *C. thermophilum* 80S ribosome sample at 200 nM concentration was applied on holey carbon grids (Quantifoil R2/1 grid, Quantifoil Micro Tools, GmbH) and plunged-frozen into liquid ethane using a Vitrobot (FEI). The Vitrobot environment chamber was programmed to maintain a temperature of 4 °C and 100% humidity. Cryo-EM data of 30 °C puromycin-treated ribosomes (flooded with 60× molar excess of *C. thermophilum* ribosome-associated molecular chaperone Ssb, overexpressed and purified from *E. coli* as described^73^) were collected at the EMBL Heidelberg facility (funded by iNEXT-Discovery) on a Titan Krios electron microscope (FEI) operating at 300 kV. Data were collected on a Quantum-K2 detector using counting mode. The *Ct*80S particles were acquired at a nominal magnification of ×165,000, with a total dose of 32.5 e^–^/Å^2^. Defocus range was set from −0.8 to −2.0 and every movie was fractioned into 40 frames. Cryo-EM data of 30 °C and 50 °C puromycin-treated ribosomes were collected on an in-house Titan Krios (FEI) operating at 300 kV. Data were collected on a Quantum-K3 detector using counting mode. The *Ct*80S particles were acquired at a nominal magnification of ×81,000, with a total dose of 41.5e^–^/Å^2^. Defocus range was set from −0.8 to −2.5 and every movie was fractioned into 149 frames.

### Single-particle analysis and model building

A total of 15,378 micrographs were used for the *Ct*80S (30 °C puromycin-treated, flooded with 60x molar excess of Ssb for eventual localization) structure determination. The frames were aligned and summed using MotionCor2 whole-image motion correction software^74^. CTFFIND4 was used for contrast transfer function (CTF) estimation of unweighted micrographs^75^. Particle auto-picking was performed with Relion 3.1^76^ (Laplacian-of-Gaussian detection) and inspected manually where majority miss-picked particles or contaminants were removed. Later, particles were extracted (480 × 480 pixels), downsampled (120 × 120 pixels) and subjected to three rounds of reference-free 2D classification in Relion 3.1. First cycles of 2D classification were performed with large search range (20 pixels) to achieve the best possible centering of the particles. The later rounds were performed in higher precision on full-size particles with smaller search ranges (10 and 5 pixels). Only properly centered class averages were selected for subsequent processing steps. Our *C. thermophilum* 80S ribosome sample suffered from a strong preferred orientation. As an alternative approach to Relion package, we tried cisTEM (correlation-based 3D reconstruction) software^77^. cisTEM was able to overcome the problem of the preferred orientation and was used for further processing. The stack of 538,868 particles from 2D classification was downsampled again (120 × 120 pixels), imported to cisTEM and auto-refined using a yeast 80S ribosome as a reference (low pass filtered to 30 Å). Still downsampled, auto-refined particles were subjected to 3D classification, which resulted in removal of 143,034 (26.52%) poorly defined particles. The remaining 395,834 particles were subjected to another round of 3D classification using less downsampled particles (240 × 240 pixels) and resulted in two useful classes clearly separating two different states of the ribosome. Particles from each class were re-extracted in full-size and auto-refined. The translocation-intermediate (TI)-POST class was subjected to additional focused classification^78,79^ (focusing on eEF2) and resulted in a new class containing eEF2 (33.45% of particles) (Supplementary Fig. 1). Processing of the 30 °C and 50 °C puromycin-treated ribosomes was performed using the same pipeline. The final resolution was measured by FSC at 0.143 value as implemented in cisTEM.

The local resolution variations were calculated with ResMap^80^. The model of *Ct*80S was initially built based on a previously published yeast structure (PDB ID: 4v88)^1^ with some proteins being built by homology modelling using the SWISS-MODEL Workspace^81^. Sequences of *C. thermophilum* proteins were assigned by BLAST^82^ and the *C. thermophilum* genome resource^34^. The models were manually built and corrected in Coot^83^. As amino acid side-chain densities and nucleotides were clearly resolved, real-space refinement was efficiently used for model building in Phenix^84^. Magnesium ligands were built as hex-aqua complexes according their size in the EM-density and when present as validated osmium-hexamine complexes in a *Sc*80S X-ray structure^1^. Octahedral geometry of the ligand complexes was set free only in the last round of refinement. Atomic models were validated using Phenix and MolProbity^85^.

### Measurement of rotations and figure preparation

40S subunit rotation and head swiveling were measured in UCSF Chimera^86^ using the command ‘measure rotation‘. For the 40S body, the structures were aligned on 26S rRNA, and the rotation between a pair of 18S rRNA (1–1150; 1620–1796) was measured. For the 40S head, the structures were aligned on the 40S body, and the rotation between a pair of 18S rRNA (1150–1620) was measured. Figures were prepared in GraphPad Prism, Pymol, UCSF Chimera and UCSF ChimeraX^87^.

### Reporting summary

Further information on research design is available in the Nature Research Reporting Summary linked to this article.

## Supplementary information

Supplementary Information

Reporting Summary

## Data Availability

The data that support this study are available from the corresponding author upon reasonable request. Cryo-EM maps have been deposited in the Electron Microscopy Data Bank under accession codes EMDB: EMD-12976 for idle POST and EMDB: EMD-12977 for (TI)-POST conformational states. The atomic models have been deposited in the Protein Data Bank under accession numbers PDB 7OLC and PDB 7OLD.
