# Supplementary material for: High-resolution structures of a thermophilic eukaryotic 80S ribosome reveal atomistic details of translocation
Source: Nat Commun. 2022 Jan 25;13:476. doi: 10.1038/s41467-022-27967-9 (PMC8789840; doi:10.1038/s41467-022-27967-9)
Supplement: Supplementary file 1 — Supplementary Information [file 41467_2022_27967_MOESM1_ESM.pdf]

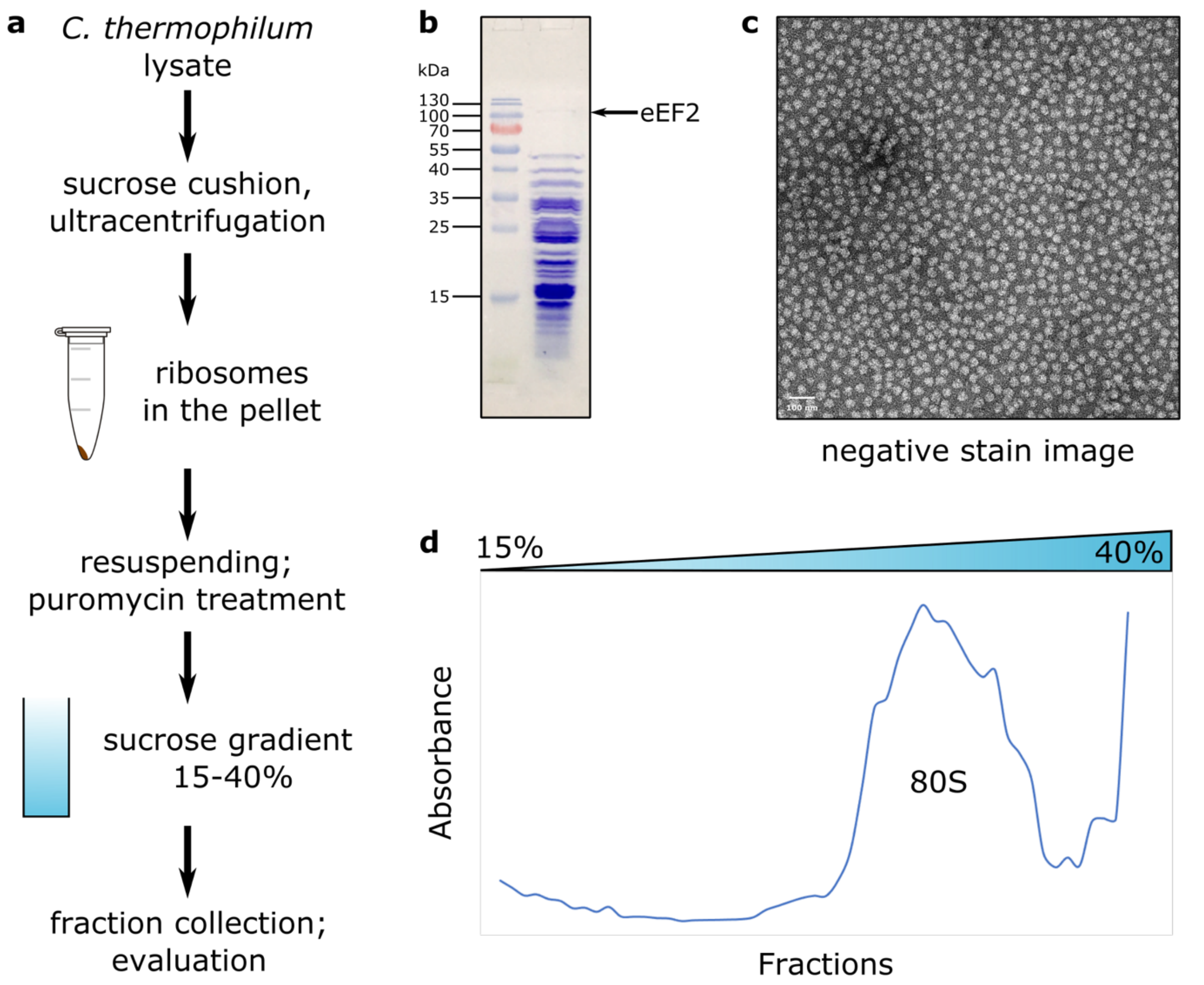

Supplementary Fig. 1 | *Chaetomium thermophilum* 80S sample preparation and quality evaluation. a, Purification scheme explaining all main steps of *Ct*80S sample preparation. **b**, SDS-PAGE of the purified *Ct*80S sample (Coomassie staining). SDS-PAGE analysis was performed after every purification. **c**, A representative negative stain EM image of the *Ct*80S sample at 80 nM concentration (a total of 5 micrographs were collected). **d**, Absorbance measurements at 260 nm of the sucrose gradient fractions indicating the presence of mostly monomeric *Ct*80S particles and some remaining polysomes in the pellet (40%).

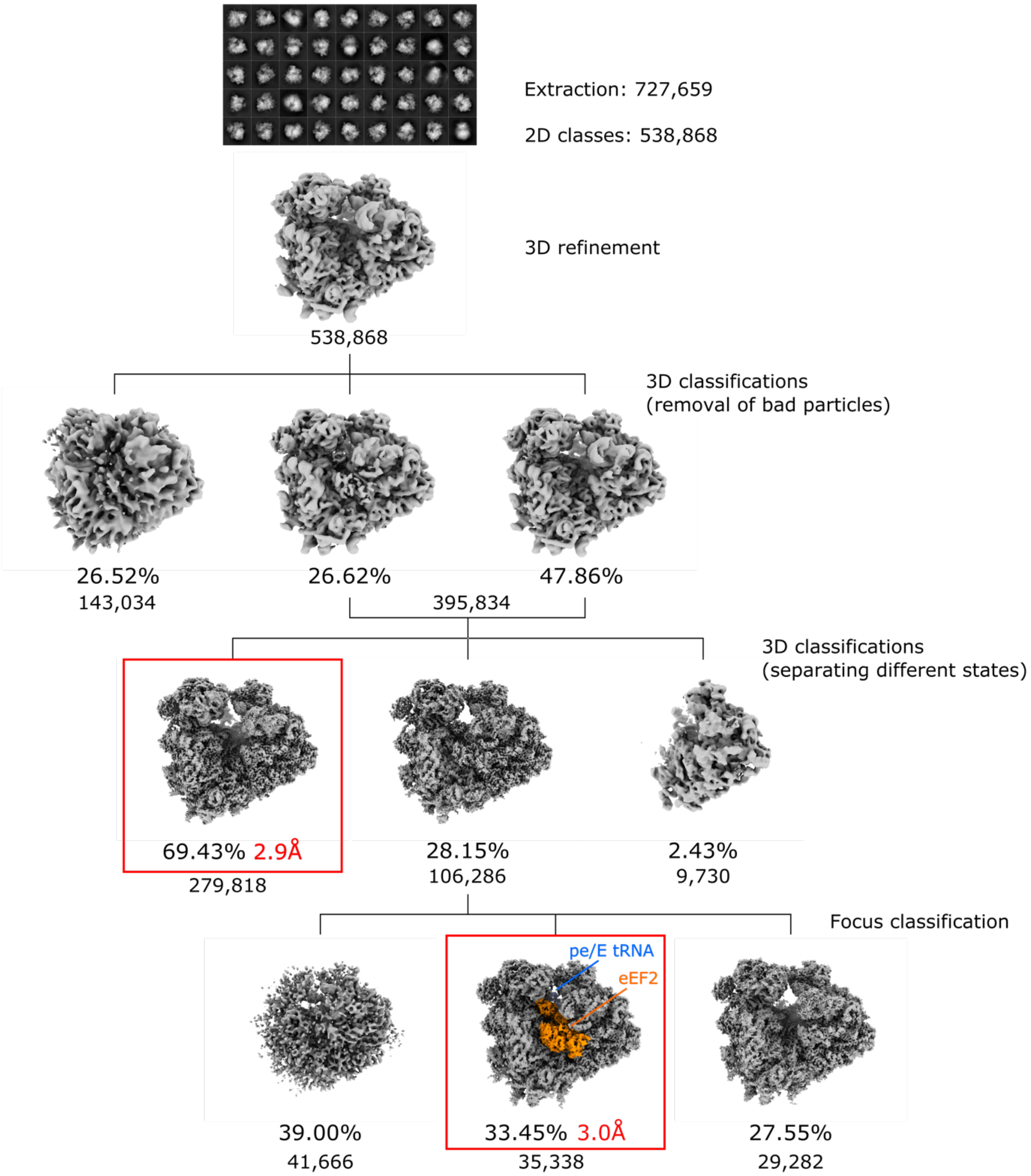

Supplementary Fig. 2 | Schematic representation of the cryo-EM single-particle analysis workflow. The main steps include frame alignment and contrast transfer function determination, 2D classification, initial 3D refinement, 3D classification and the independent refinement of selected classes. Focused 3D classification was performed for the (TI)-POST state using a mask focused on eEF2 (orange).

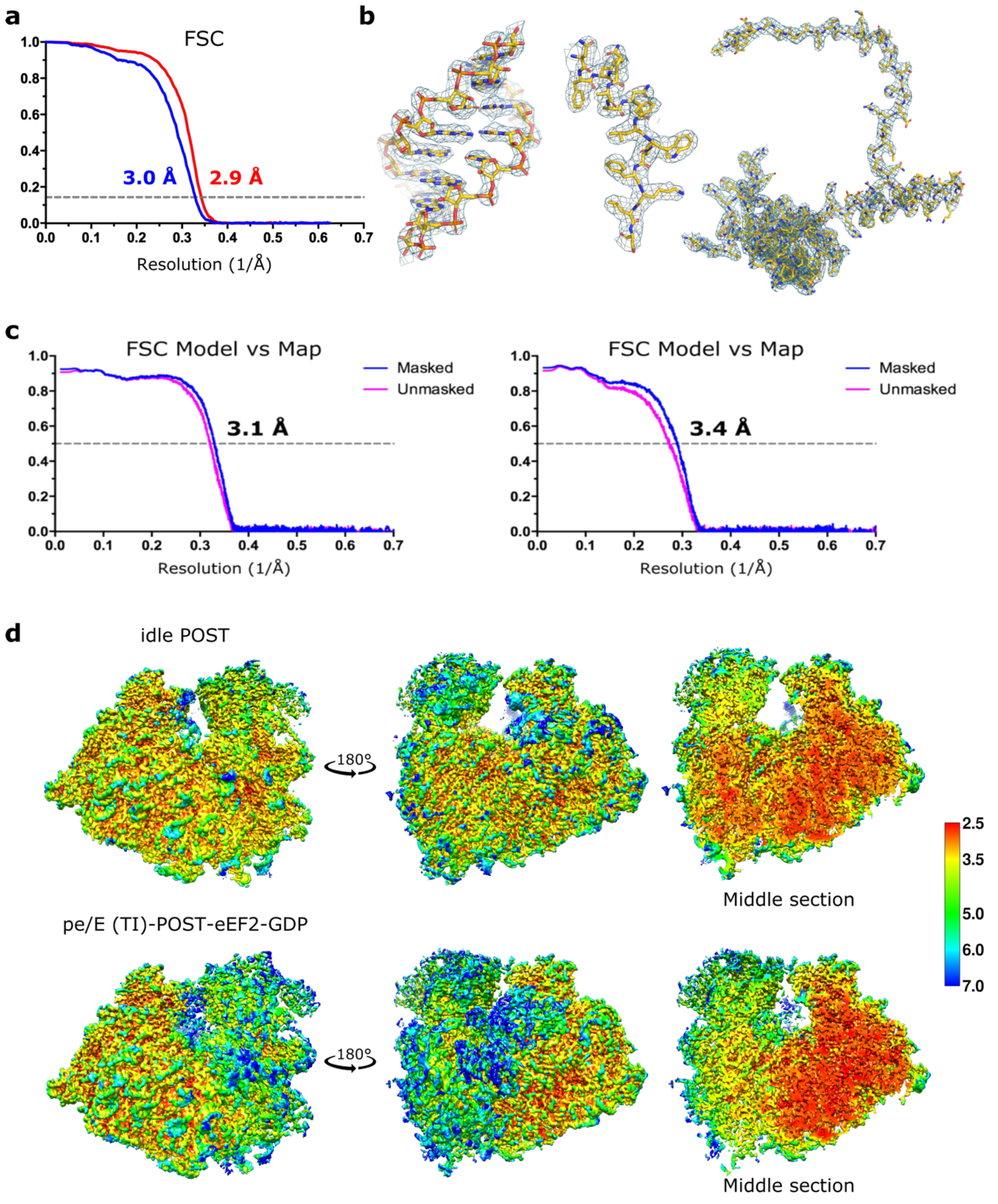

Supplementary Fig. 3 | Cryo-EM analysis of *Ct*80S ribosomes. a, Fourier shell correlation curves for the final models of the idle POST (red) and (TI)-POST (blue) states. **b**, Representative cryo-EM maps of segments from 26S rRNA and uL30 and a whole ribosomal protein eL21. **c**, FSC curves of model vs. map calculated by Phenix. **d**, Surface representation and middle section of the cryo-EM maps of the idle POST and (TI)-POST states color coded according to local resolution as indicated.

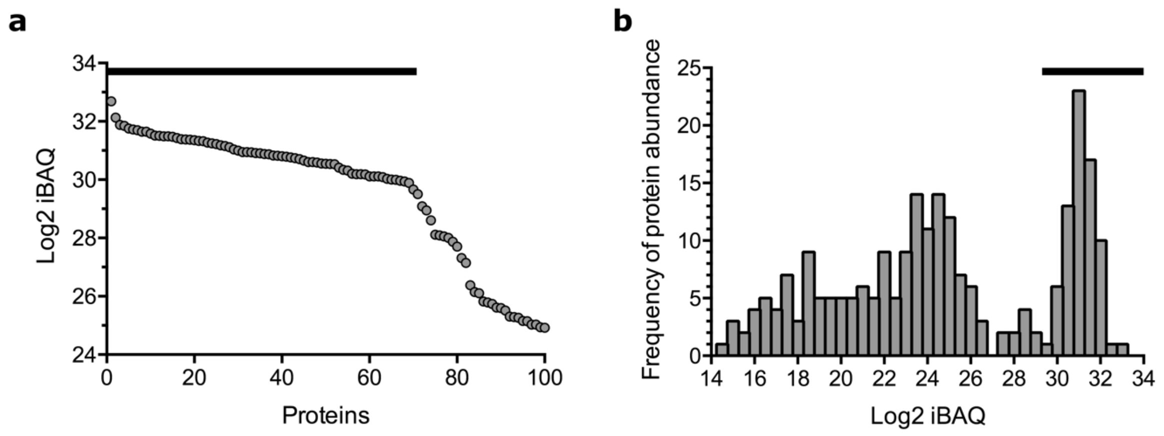

Supplementary Fig. 4 | Mass spectrometric analyses using intensity based absolute quantification (iBAQ). Overall, 240 proteins were identified in three independent analyses and quantified using intensity based absolute quantification. A population of 69 highly abundant proteins with Log2 iBAQ > 30 (marked with black line) are detected, whereas the bulk of proteins is less abundant of by a factor of more than 10. Between the population of high abundant proteins and low abundant proteins there is a small population of 17 proteins. **a**, Protein abundances in preparations of purified *Ct*80S. **b**, Frequency of protein abundances in preparations of purified *Ct*80S.

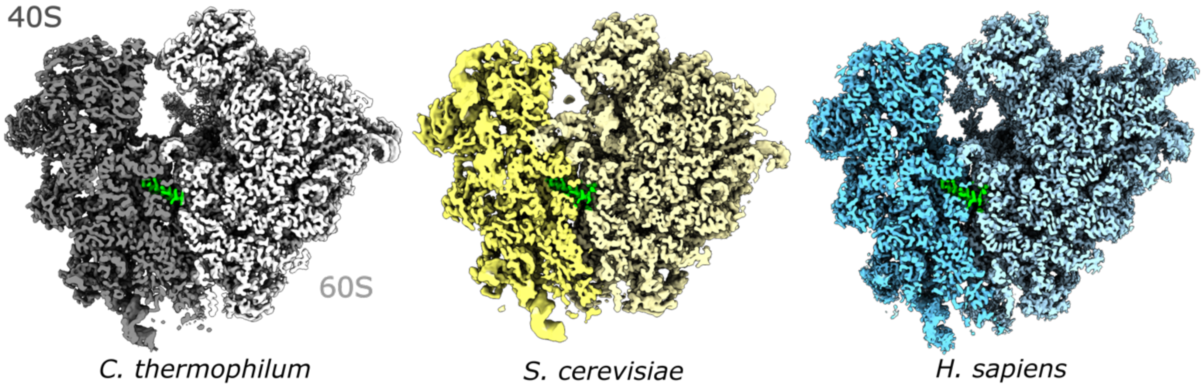

Supplementary Fig. 5 | Cryo-EM densities for canonical eL41 in 80S ribosomes from different organisms. The location of eL41 (green) in the interface between 40S and 60S subunits is conserved in *C. thermophilum* 80S (this study), *H. sapiens* 80S (PDB ID: 6ek0)[1] and *S. cerevisiae* 80S (PDB ID: 4v88)[2].

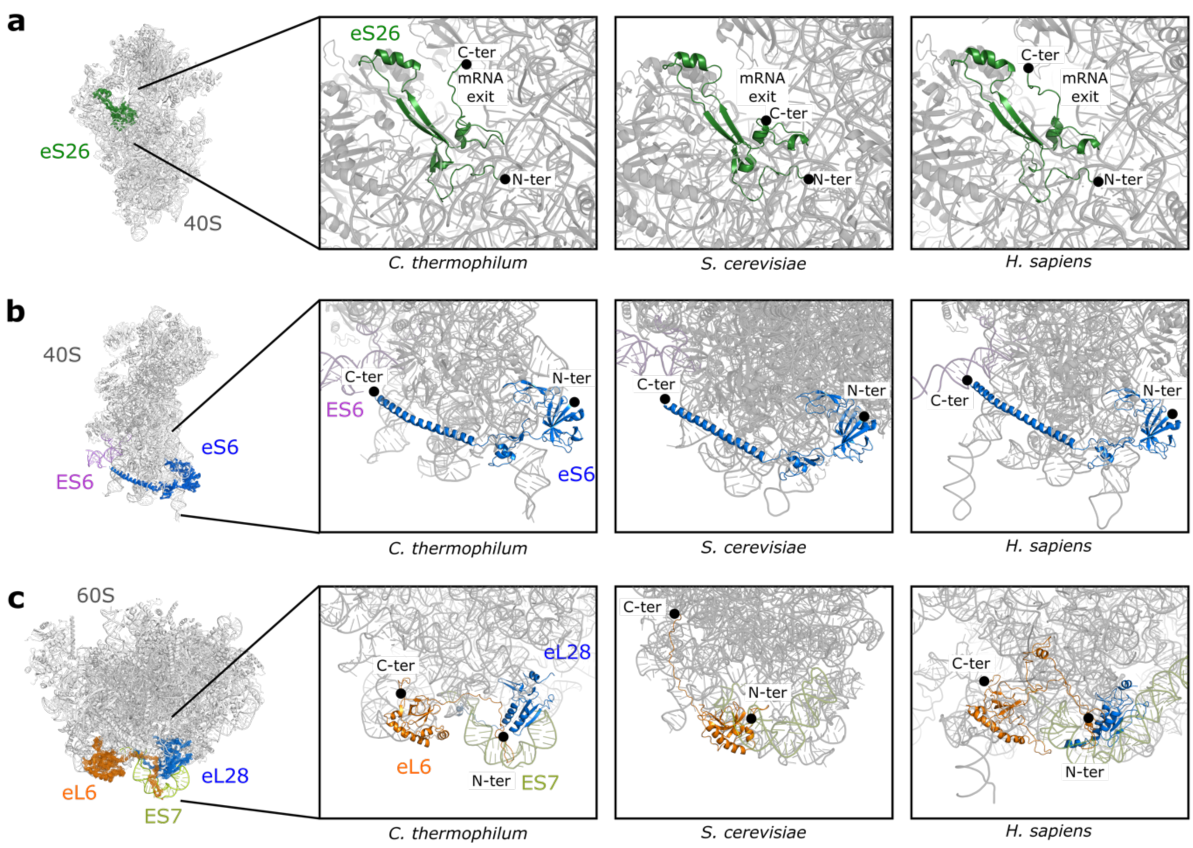

Supplementary Fig. 6 | Ribosomal proteins eS6, eS26, eL6 and eL28. Left panels show overviews for the positions of RP (**a**) eS26 (green), (**b**) eS6 (blue), and (**c**) eL6 (orange) and eL28 (blue). **a**, Comparison of the eS26 C-terminal segment in *C. thermophilum, S. cerevisiae* (PDB ID: 4v88)[2] and *H. sapiens* (PDB ID: 6v6x)[3]. The C-terminal extensions of *C. thermophilum* and *H. sapiens* eS26 reach into the mRNA-exit channel. **b**, Comparison of the eS6 C-termini in *C. thermophilum, S. cerevisiae* and *H. sapiens*. In *C. thermophilum* the α-helical C-terminus is similar in length to *S. cerevisiae*, but extends closer to ES6, while *H. sapiens* eS6 forms contacts with ES6. **c**, Comparison of eL6, eL28 and ES7 in *C. thermophilum, S. cerevisiae* and *H. sapiens.* eL28 is not present in *S. cerevisiae*, while in *C. thermophilum* eL6 and eL28 interact with ES7, thus like in *H. sapiens* stabilizing ES7 on the ribosomal surface. In all panels RP termini are labelled and highlighted by black dots.

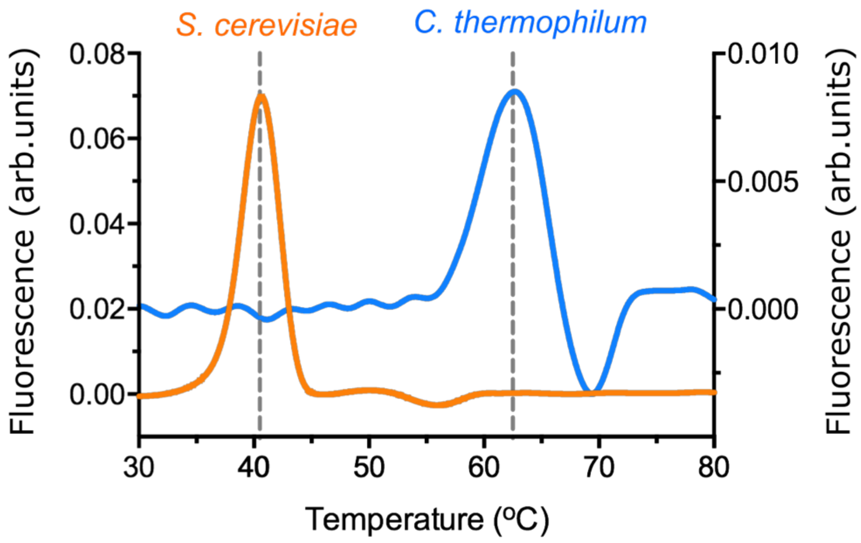

Supplementary Fig. 7 | Melting curves (1st derivative) for *S. cerevisiae* and *C. thermophilum* 80S ribosomes. *Ct*80S has a 22 °C higher melting temperature (blue) when compared with *S. cerevisiae* (orange) (T_m of 62.5 °C versus 40.5 °C).

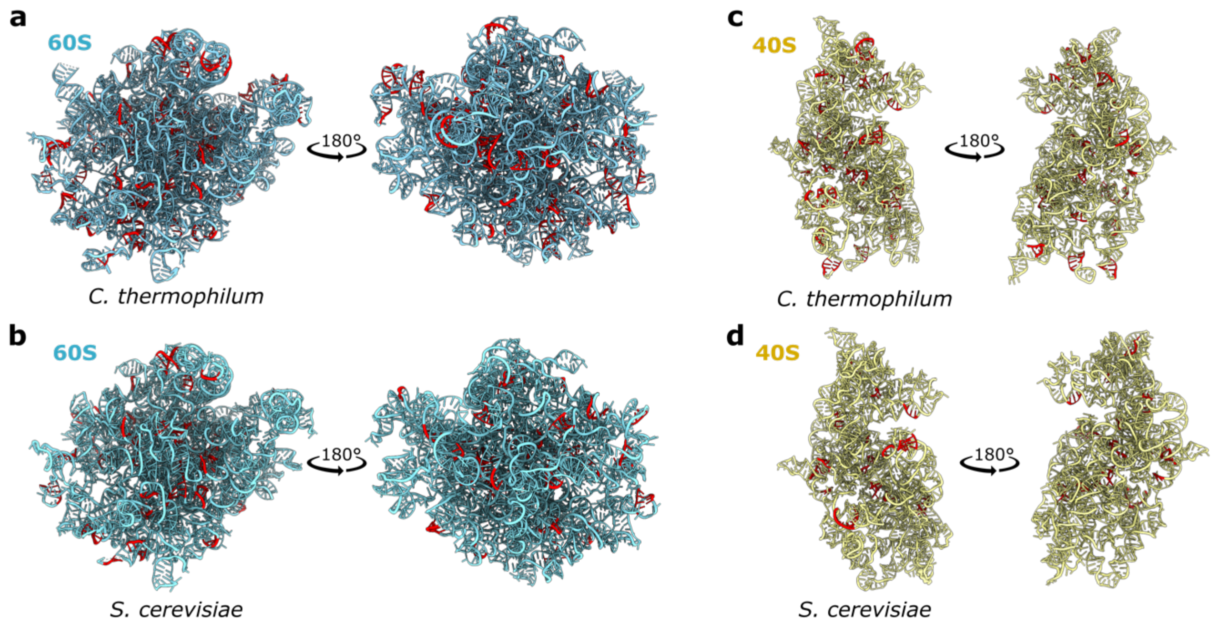

Supplementary Fig. 8 | G and C homoiterons in the 26S and 18S rRNA of *S. cerevisiae* and *C. thermophilum.* Homoiterons (red) in the rRNA of *C. thermophilum* large ribosomal (**a**) and small ribosomal subunits (**b**). Homoiterons (red) in the rRNA of *S. cerevisiae* (PDB ID: 4v88)[2] large ribosomal (**c**) and small ribosomal subunits (**d**).

Secondary Structure: large subunit ribosomal RNA - 5' region

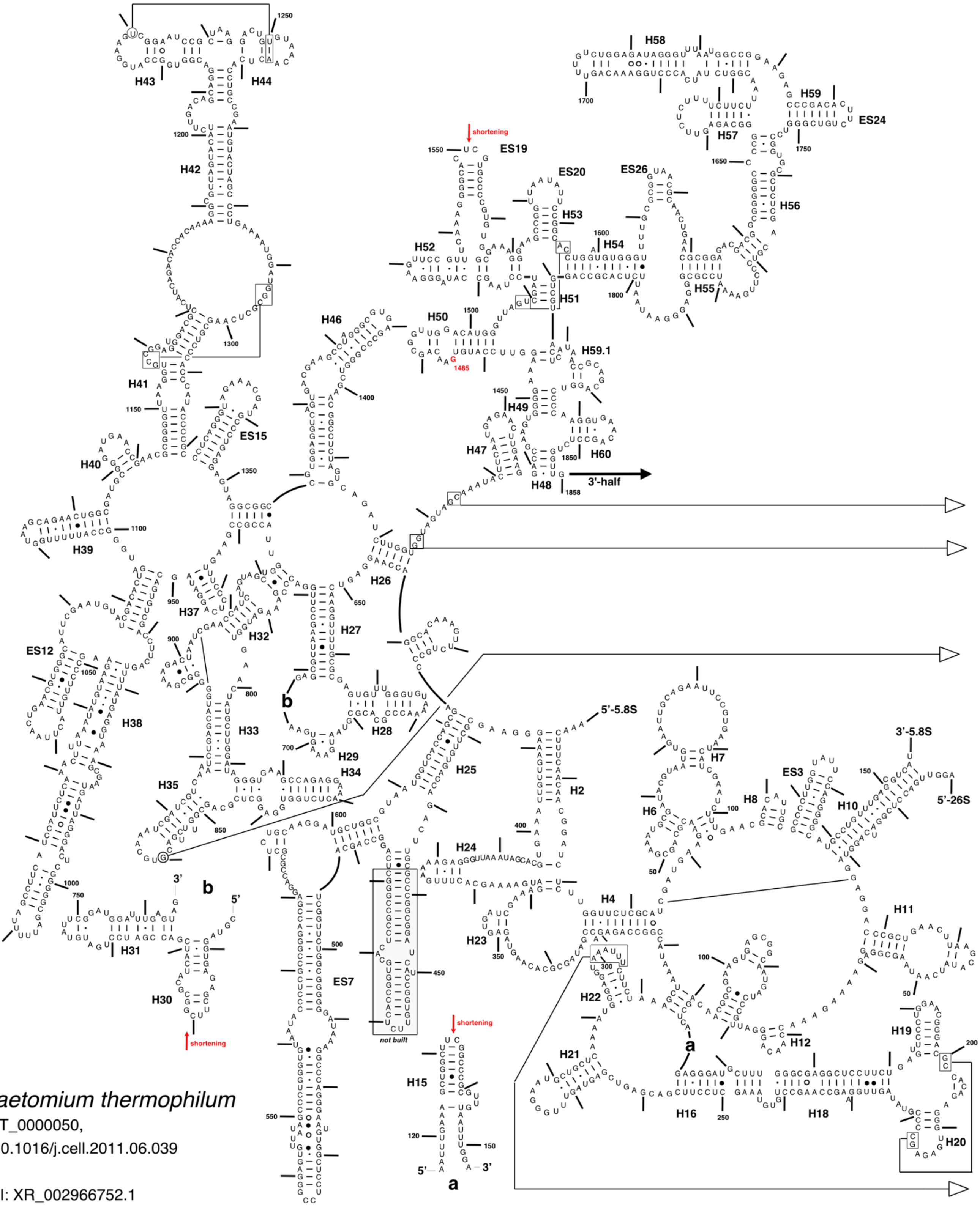

Chaetomium thermophilum

CTHT_0000050,

doi:10.1016/j.cell.2011.06.039

NCBI: XR_002966752.1

Secondary Structure: large subunit ribosomal RNA - 3' region

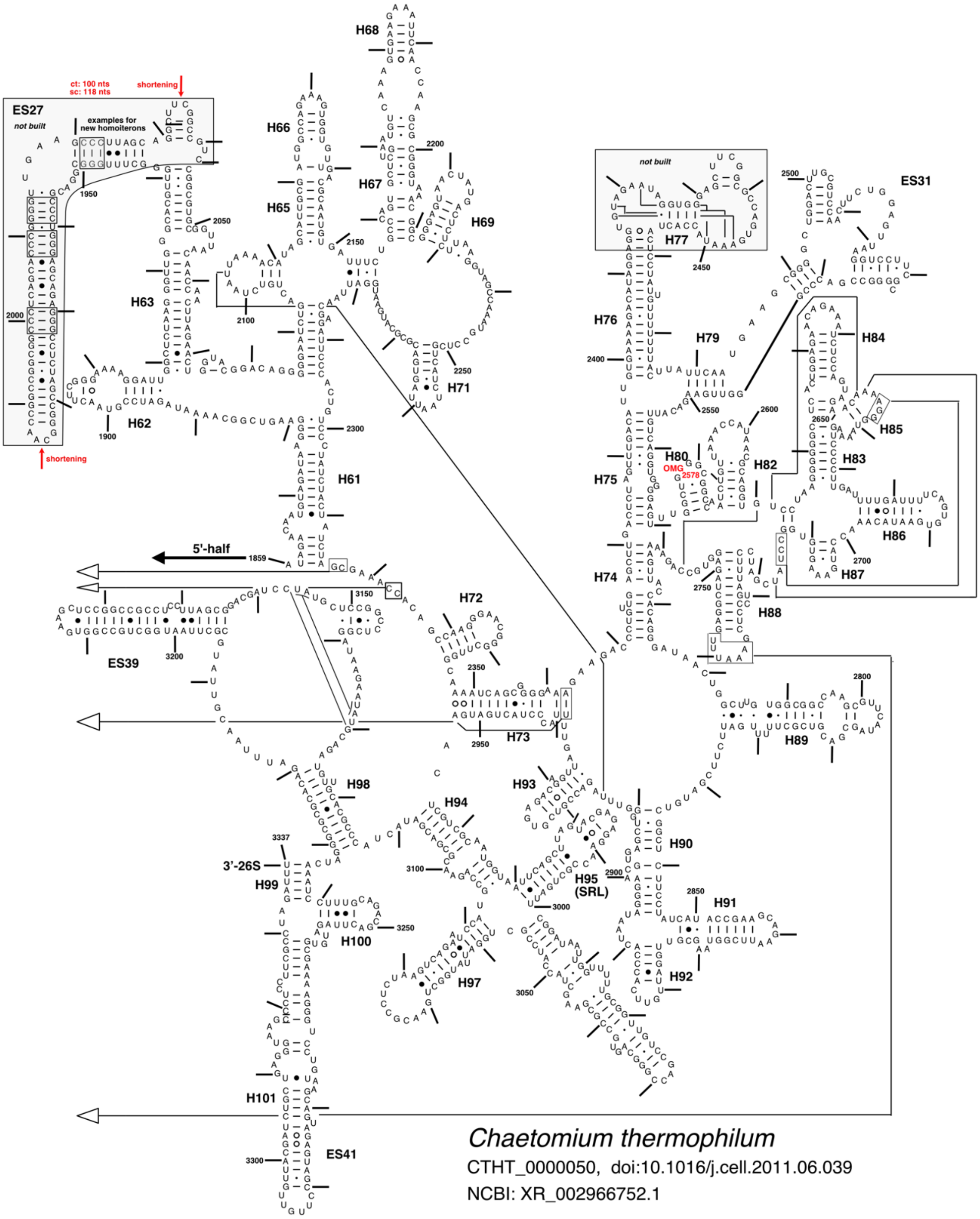

Chaetomium thermophilum

CTHT_0000050, doi:10.1016/j.cell.2011.06.039

NCBI: XR_002966752.1

Secondary Structure: small subunit ribosomal RNA

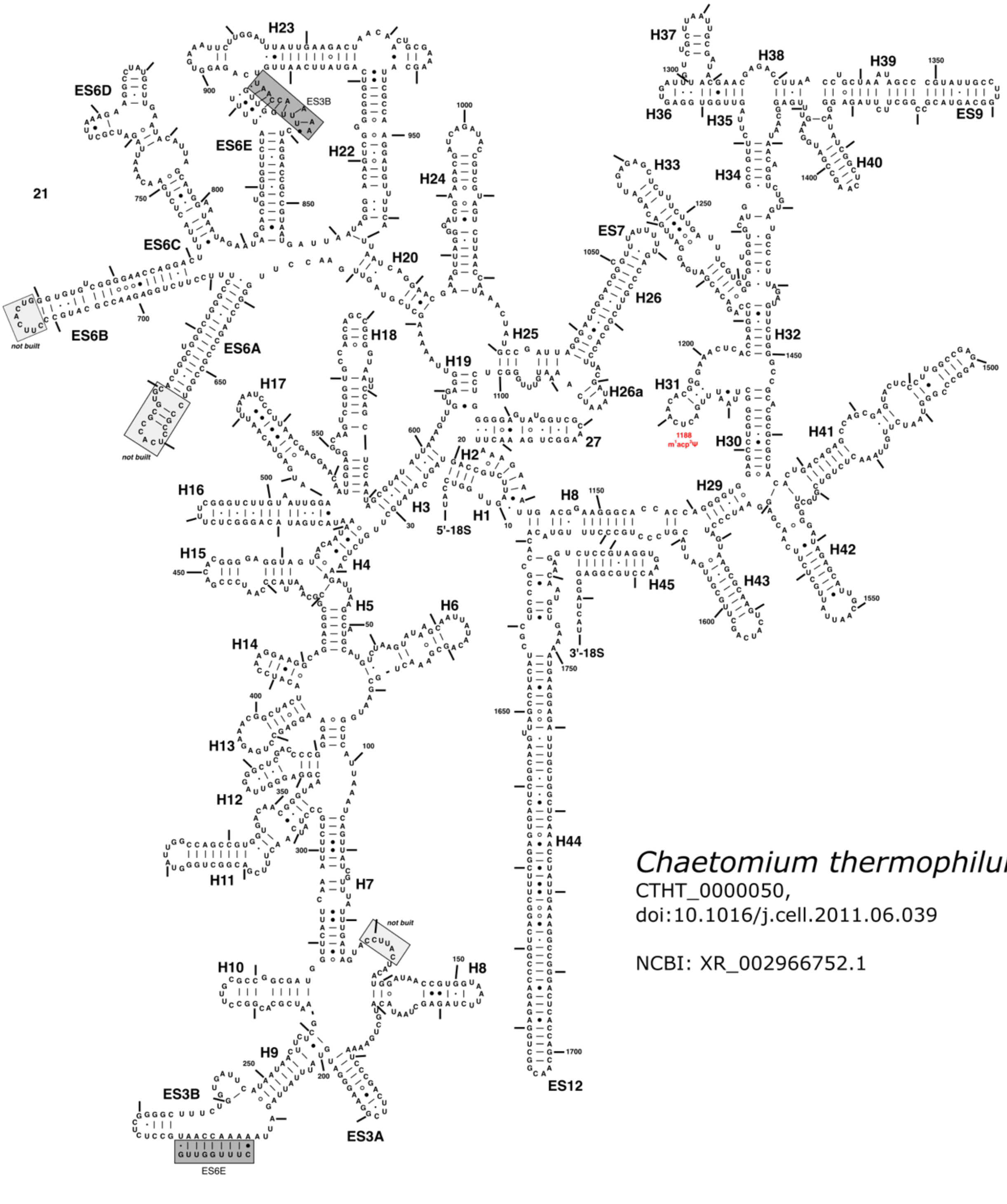

Chaetomium thermophilum

CTHT_0000050,

doi:10.1016/j.cell.2011.06.039

NCBI: XR_002966752.1

Secondary Structure: 5S ribosomal RNA

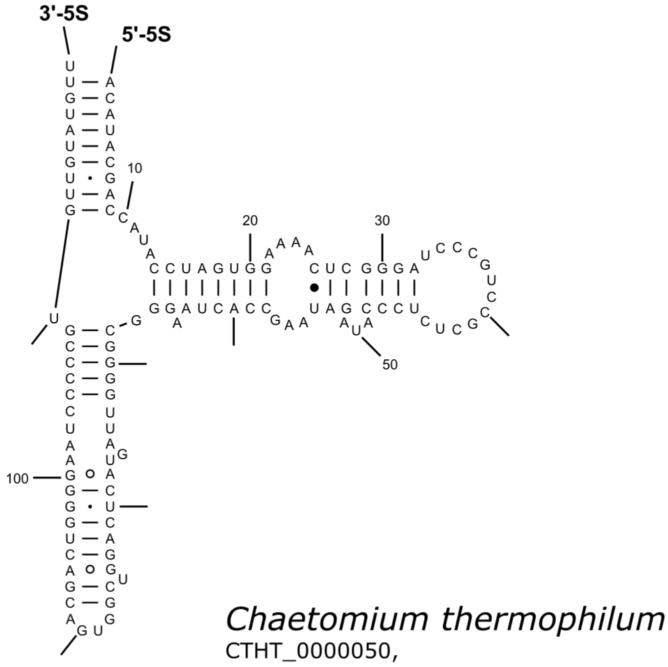

Chaetomium thermophilum
CTHT_0000050,
doi:10.1016/j.cell.2011.06.039

NCBI: XR_002966752.1

Supplementary Fig. 9 | Secondary structure diagram of *C. thermophilum* rRNAs. The diagrams of *S. cerevisiae* rRNA were taken from the Comparative RNA Web (CRW) Site (https://crw-site.chemistry.gatech.edu/) and modified according to the final rRNA models. Nucleotides, helices and ES are numbered. Canonical base pairs are depicted with (-), while (•) denote GU wobble base pairs, (○) – AG base pairs, and (●) – other non-Watson-Crick base pairs. Regions that are not built in the model are marked with frames. Additional homoiterons within 26S rRNA ES27 are boxed. Modified bases and G1485 constricting the tunnel exit are highlighted in red. Shortenings of ESs compared to *Sc* rRNAs are indicated. Dark grey boxes indicate interacting segments; light grey boxes indicate areas of rRNA that were not built in our model.

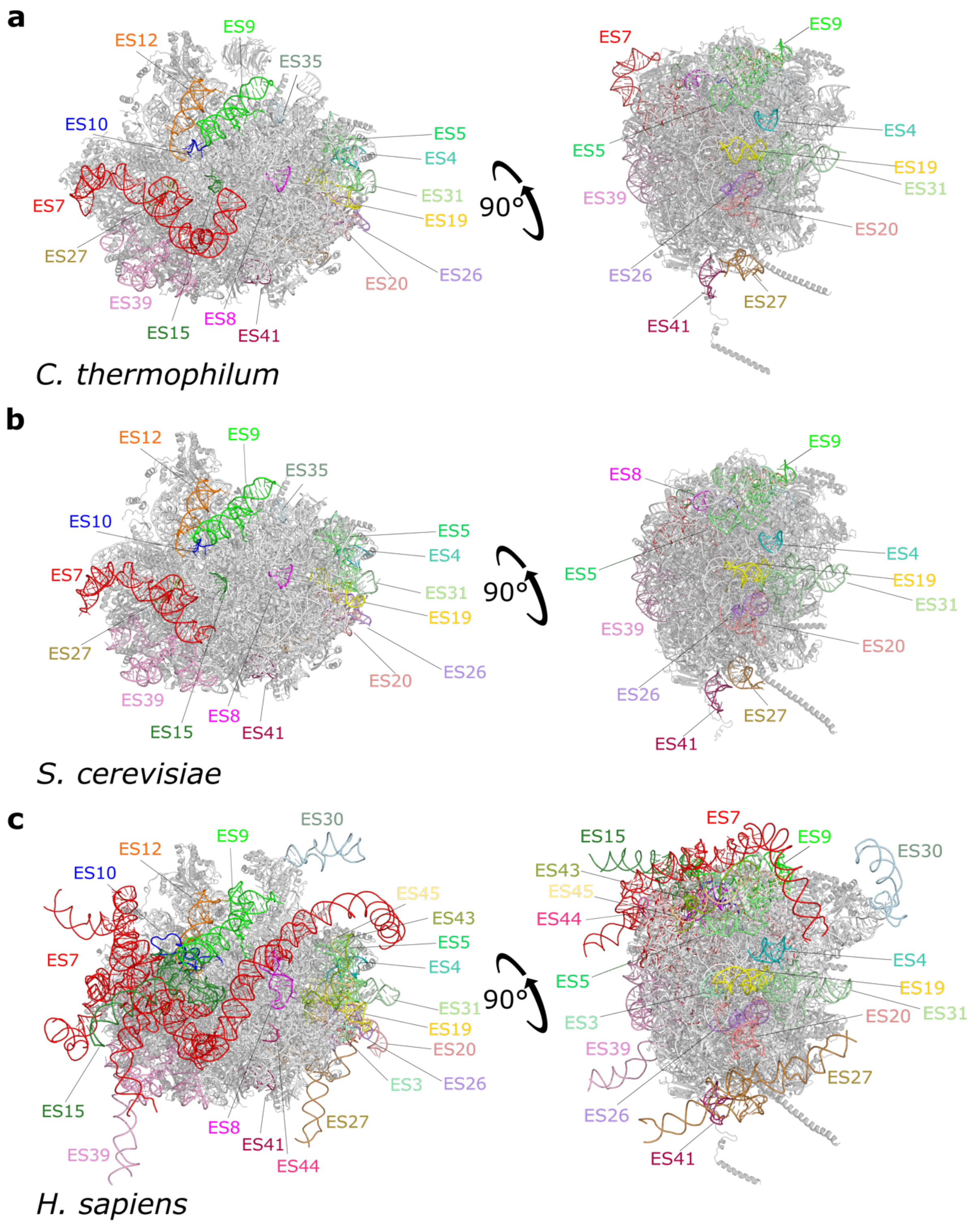

Supplementary Fig. 10 | Comparison of the 26S rRNA expansion segments. Molecular models for the 60S subunits of (**a**) *C. thermophilum* (this study), (**b**) *S. cerevisiae* (PDB ID: 4v88)[2] and (**c**) *H. sapiens* (PDB ID: 6ek0)[1] with expansion segments (ESs).

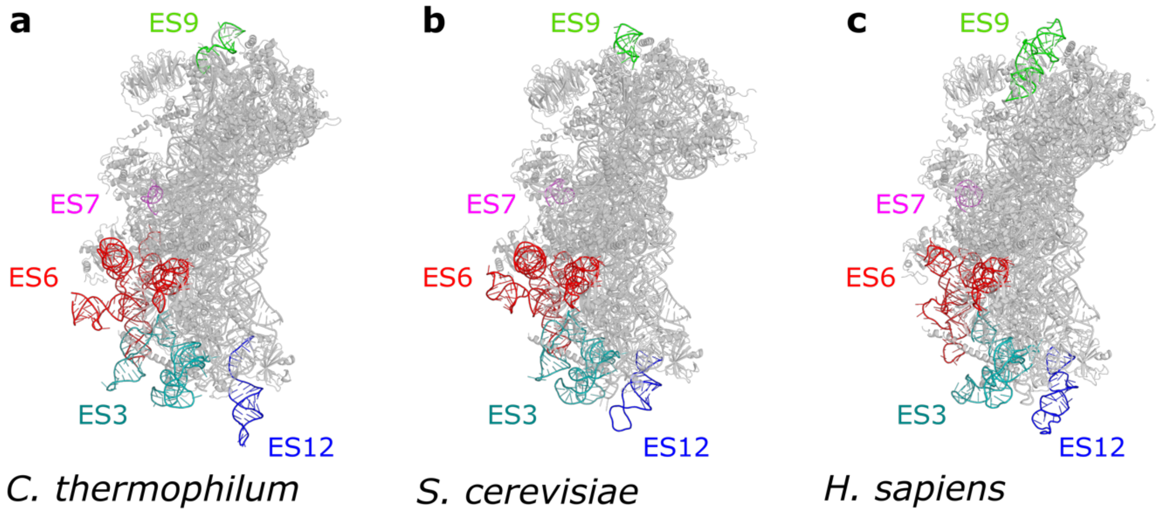

Supplementary Fig. 11 | Comparison of the 18S rRNA expansion segments. Molecular models for the 40S subunits of (**a**) *C. thermophilum* (this study), (**b**) *S. cerevisiae* (PDB ID: 4v88)[2] and (**c**) *H. sapiens* (PDB ID: 6ek0)[1] with highlighted expansion segments (ESs).

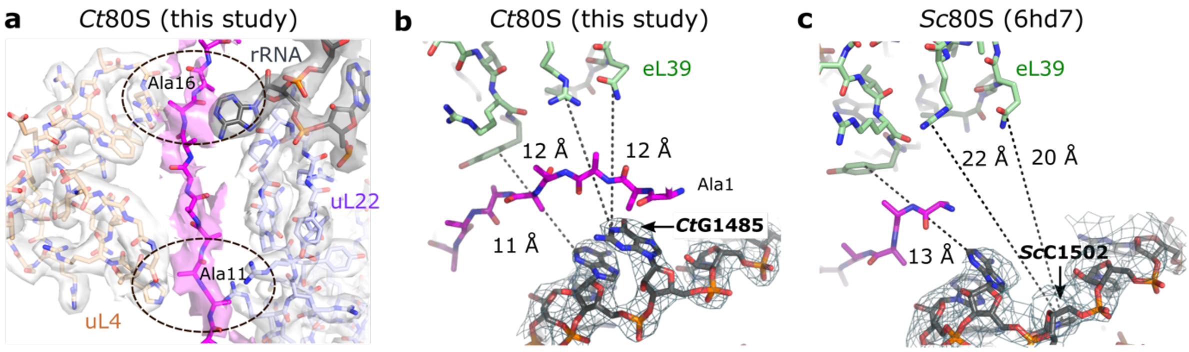

Supplementary Fig. 12 | NC interactions at the tunnel exit of the 80S ribosome. **a**, Constrictions of the exit tunnel created by the uL4 and uL22 proteins (highlighted by dashed circles). The contouring level for NC is 1.5 σ, while the rest of the proteins and rRNA are shown at 4.0 σ. **b**, **c**, Comparison of the ribosomal tunnel exit in *C. thermophilum* and *S. cerevisiae*[4]. (**b**) shows how the bulged-out *Ct*80S G1485 (contour level 4.0) significantly narrows the tunnel while *Sc*80S C1502 (contour level 5.0 σ) in (**c**) is flipped-in and the tunnel is wide open. The NC is shown in magenta and relevant distances are indicated.

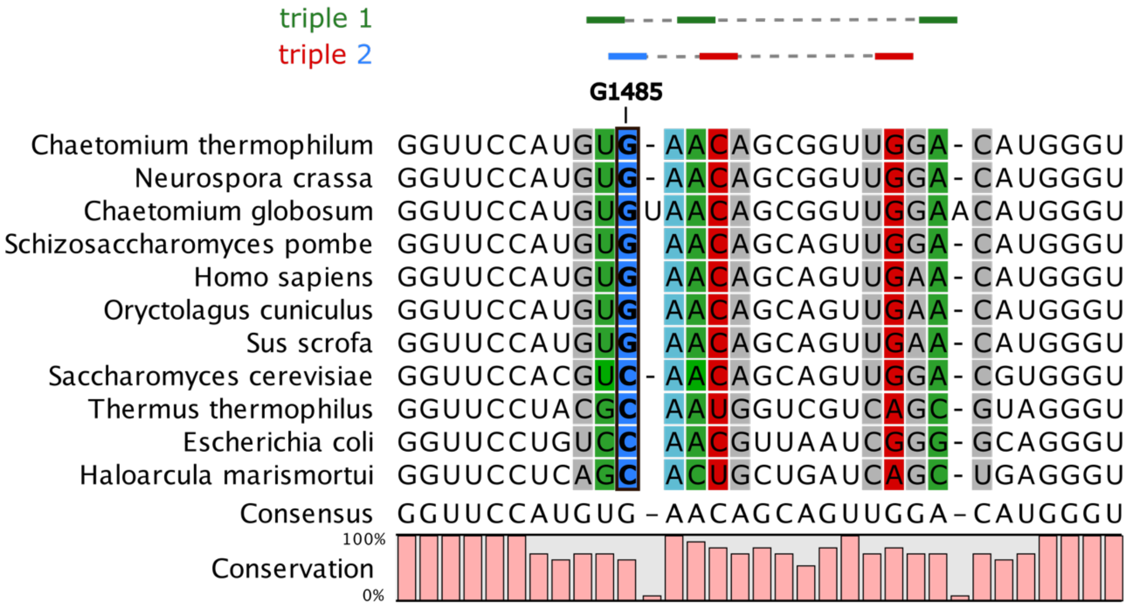

Supplementary Fig. 13 | 26S rRNA H50 sequence alignment. The 26S rRNA of *C. thermophilum* around the 'nested base-triple knot' of H50 is compared with equivalent regions of eukaryotic, bacterial and archaeal organisms. Color code for the knot is according to Figure 3. The flipping nucleotide (*Ct*G1485) and the two base triples are highlighted. Sequence consensus and overall conservation are indicated below the alignment. While all higher eukaryotes comprise a guanine base, lower eukaryotes (yeast), bacteria and archaea provide a cytosine at the equivalent position. *C. globosum* rRNA H50 contains a unique UA base-pair insertion.

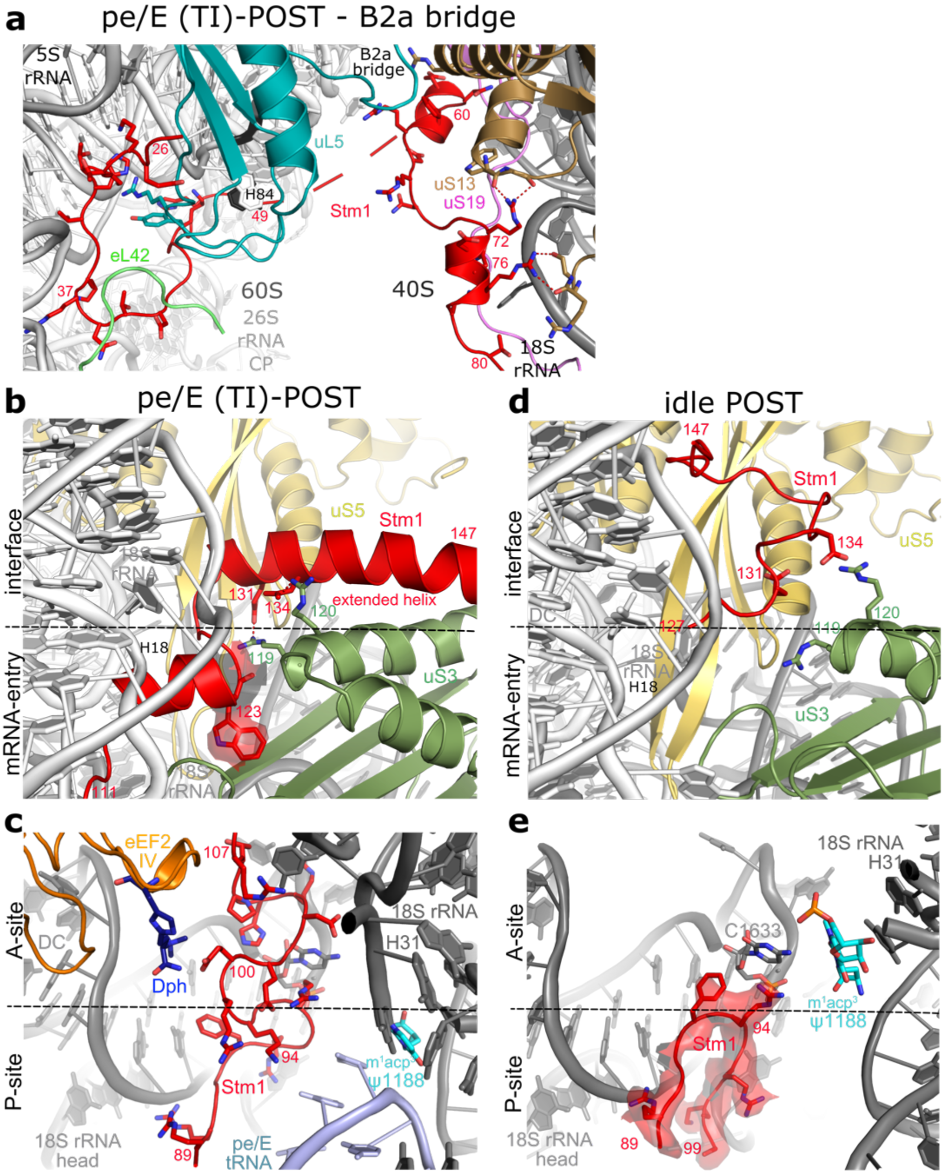

Supplementary Fig. 14 | Details of Stm1 interactions in both rotation states. Left panels are for the pe/E (TI)-POST state and right ones for the idle POST state. **a**, Stm1 stabilizes the rotated state by bridging the 60S and 40S at the B2a bridge. Bridging residues are not built. Stabilizing interactions of the Stm1 (R72, R76) with the main chain of uS13 are indicated. **b**, The mRNA-entry site is blocked by Stm1 C-terminal fragment, which the interferes with 40S head back-swiveling by intercalating between uS3 and uS5 with conserved interactions as indicated. **c**, Stm1 crawls through the A- and P-sites and around Dph of eEF2. **d**, In the idle POST state, the mRNA-entry site is free, the extended helix is dissolved and folded back on uS5. uS3 is rotated away. **e**, The A-site is empty and the Stm1 conformation in the P-site is different in the idle POST state. Representative cryo-EM densities are contoured at 3.0 σ (panel c) and 2.0 σ (panel e).

a

```
CtStm1       1 MSVASKNPFDILGNTEDE--TPVVPVKAVEKTSTHTAKRNTDGLPPKGHAAGNRRGGANVSGNEAAFRDR  68
ScStm1       1 MS----NPFDLLGNDVEDADVVVLPPKEIVKSNTSSKKADVPPPSADPSKARKNRP--RPSGNEGAIRDK  64
               MS....NPFDlLGNs.--..hsVlPsK.l.KosTpo.K.shss.sscsp.A.ppRs..psSGNEtAhRD+

                                                                         extended
CtStm1      69 NAGRDANRGKPTDEAAPRGGRRGGFRGRGKREEGDRHPTRSAPRSNSEKQASQGWGATEGEAELKDEQAA 138
ScStm1      65 TAGRRNNRSKDVTDSATTKKS-------NTRRATDRHSR--TGKTDTKKKVNQGWGDDKKELSAEKEAQA 125
               sAGRcsNRtKshs-tAsp..p.......spRc.sDRHsp..hs+osocKphsQGWGssc.EhphccE..A
                     helix
CtStm1     139 EEIAQTEKKEAAEGEAAPEAEAKEEEPQEKVLTYDQYLAKLAEKKLALEQENALKVRKPNEGAEDKFKGL 208
ScStm1     126 DAAAEI-AEDAAEAEDAGKPK-------TAQLSLQDYLNQQANNQF----------NKV-PEAKKVELDA 176
               -.hAph..c-AAEtEsAscsc.......p..LohppYLspbAppph..........pKs...Acc.bbsh

CtStm1     209 KPLTKNEDEALFAPTVQKKERQRERKTKQIIEIENRYVEERPRGGRGGRG--AR---DGARGGRGRGGAP 273
ScStm1     177 ERIETAEKEAYVPATKVKNVKSKQLKTKEYLEFDATFVESNTRKNFGDRNNNSRNNFNNRRGGRG---A- 242
               ..lppsEcEAhhssT..Kp.+p+p.KTKphlEh-sp@VEppsR.s.GsRs..tR...ss.RGGRG...A.

CtStm1     274 RGGRGGAKENAAPAINT------NDETAFPSLGSR 302
ScStm1     243 RKGN--NTANATNSANTVQKNRNIDVSNLPSLA-- 273
               R.Gp..sp.NAhsthNT.......D.oshPSLt..
```

b

```
CtStm1       1 M----------SVASK---------NPFDIL-----------GNTE------------------------  16
HsSREBP1     1 MPGHLQEGFGCVVTNRFDQLFDDESDPFEVLKAAENKKKEAGGGGVGGPGAKSAAQAAAQTNSNAAGKQL  70
               M.........sVhs+.........sPF-lL..........Gss........................

CtStm1      17 ------DETPVVPVKAVEKTSTHTAKRNTDGLPPKGHAAGNRR---GGANVSGNEAAFRDRNAGRDANRG  77
HsSREBP1    71 RKESQKDRKNPLPPS-----VGVVDKKEETQPPVALKKEGIRRVGRRPDQQLQGEGKIIDRRPERRPPRE 135
               ......DcpsslPsp.....sshhsK+pps..Ps..+..G.RR....ssp...sEt.h.DRps.RcssR.

CtStm1      78 K------------------PT---------DEAAPRGGR-----RG-GFRGRGKREEGDRHPTRSAPRS 113
HsSREBP1   136 RRFEKPLEEKGEGGEFSVDRPIIDRPIRGRGGLGRGRGGRGRGMGRGDGFDSRGKREF-DRHSGS--DRS 202
               +..................Ph.........sbt.sRGGR.....RG.GFctRGKREb.DRHssp..sRS
                                                  extended            helix
CtStm1     114 NSEKQ------------ASQGWGATEGEAELKDEQAAEEIAQ--------TEKKEAAEGEAA-P----E- 157
HsSREBP1   203 SFSHYSGLKHEDKRGGSGSHNWGTVKDELTESPKYIQKQISYNYSDLDQSNVTEETPEGEEHHPVADTEN 272
               s.p+b...........tSpsWGhhcsEhpbpscbh.cpItb........s.pcEhsEGE.h.P....E.

CtStm1     158 -----AEAKEEEPQEKVLTYDQYLAKLAEKKLALEQENA---LKVRKPNEGAEDKFKGLKPLTKNEDEAL 219
HsSREBP1   273 KENEVEEVKEEGPKEMT--LDEW-------KAIQNKDRAKVEFNIRKPNEGADGQWKKGFVLHKSKS--- 330
               ......EhKEE.PpEbh..hDp@.......Khhbpp-pA...hplRKPNEGA-sp@K..bsLhKscs...

CtStm1     220 FAPTVQKKERQRERKTKQIIEI----ENRYVE-ERPRGGRGGRGARDGARGGRGRGGAPRGGRGGAKEN- 283
HsSREBP1   331 -EEAHAEDSVMDHHFRKPANDITSQLEINFGDLGRP--GRGGRG----GRGGRGRGGRPN--RGSRTDKS 391
               ...hh.ccp.bcc+bpK.h.-I....E.p@s-..RP..GRGGRG....tRGGRGRGG.Pp..RGt.p-p.

CtStm1     284 AAPAINTNDETAFPSLGSR 302
HsSREBP1   392 SASAPDVDDPEAFPALA-- 408
               tAsA.shsD.pAFPtLt..
```

Supplementary Fig. 15 | *Ct*Stm1 sequence alignments. *Ct*Stm1 pairwise sequence alignment[5] with the yeast (**a**) and human (**b**) homolog. Legend: bold type, included in model in (TI)-POST state; blue, α-helix; green, conserved regions with RG-repeats; orange box, 94-RGR region; red, conserved WG-motif; magenta, other conserved interactions with RPs. Conservation (in italics): l, aliphatic; @, aromatic; h, hydrophobic; o, alcoholic; p, polar; t, tiny; s, small; b, bulky; +, positively charged; -, negatively charged; c, charged.

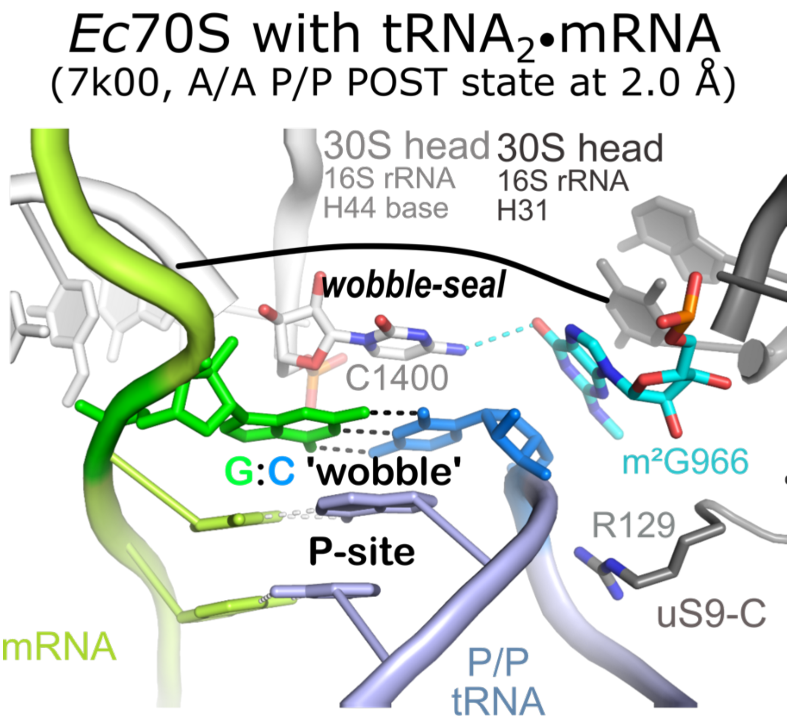

Supplementary Fig. 16 | The *wobble-seal* in the bacterial P-site. Bacteria like *E. coli* have an alternative rRNA modification (m^2G) in the P-site[6] that allows for *wobble-seal* formation.

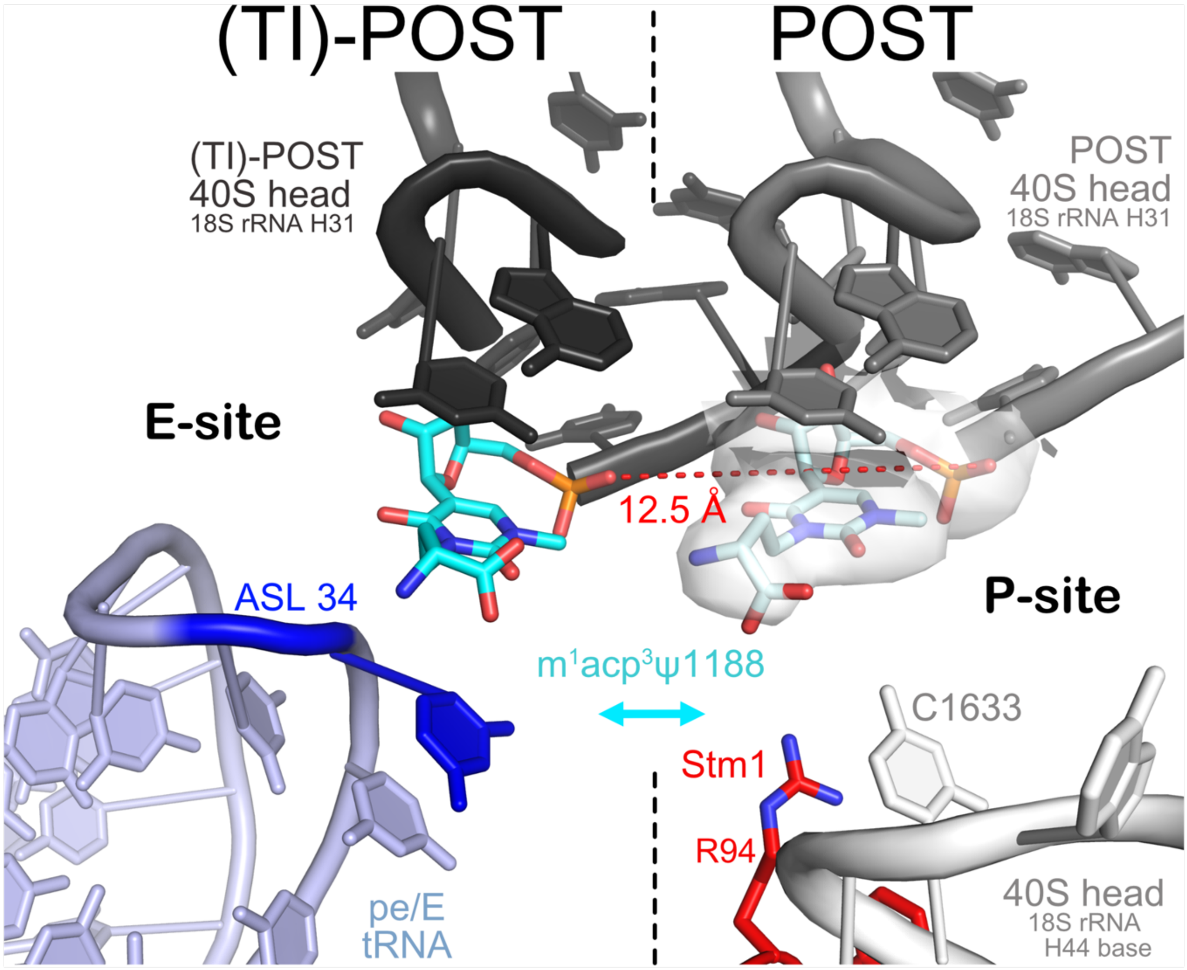

Supplementary Fig. 17 | Dynamics of the m^{1}acp^{3}Ψ1188 hyper-modification during 40S head swiveling. Superposition of pe/E (TI)-POST and idle POST states of *Ct*80S (based on 60S) zoomed into P- and E-sites (selected view, states separated by dashed line: E-site shown for (TI)-POST state and P-site shown for POST state). The hyper-modified base follows the swiveling of the 40S head (dashed line and arrow), exactly matching the tRNA-ASL distance between the P-site (mRNA position occupied by Stm1 R94 in the idle POST state) and the E-site (occupied by pe/E tRNA in the (TI)-POST state). Cryo-EM map is shown at a 2 σ level.

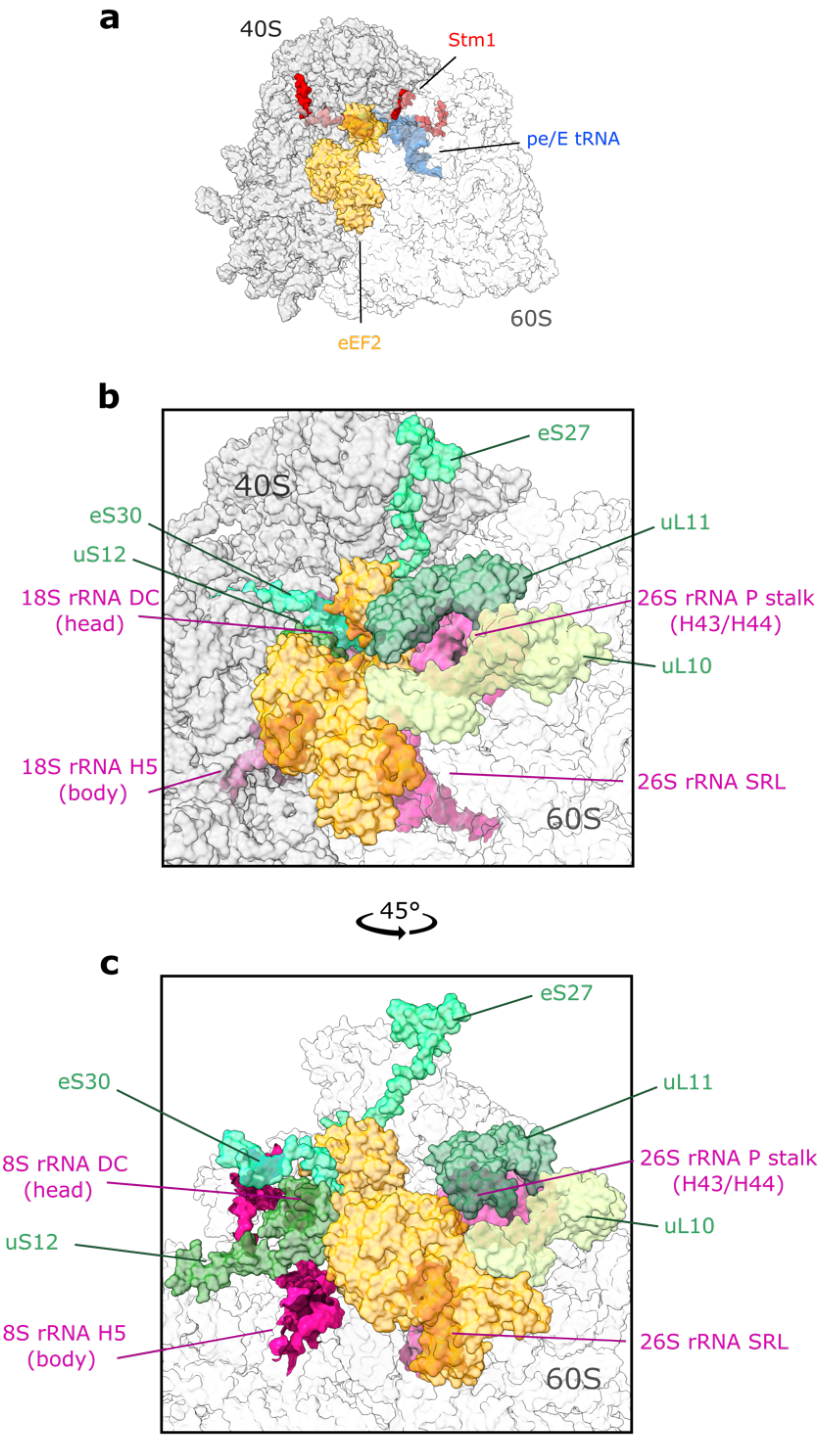

Supplementary Fig. 18 | eEF2 interactions with *Ct*80S in the (TI)-POST state. a, Surface representation of the *Ct*80S (TI)-POST state with Stm1 shown in red, tRNA in blue and eEF2 in pale orange. **b**, Zoom into the eEF2 interactions with ribosomal proteins (different shades of green) and rRNA (magenta). **c**, Rotated view that highlights the eEF2 interactions at the DC.

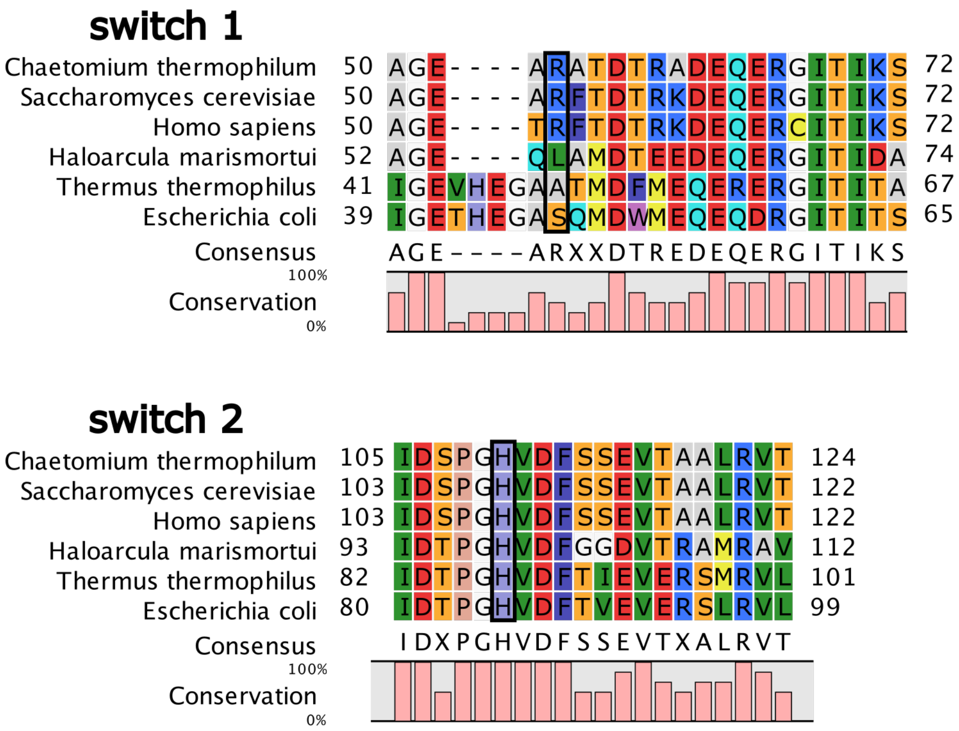

Supplementary Fig. 19 | eEF2 switch 1 and switch 2 sequence alignment. The *C. thermophilum* eEF2 sequence is compared with corresponding regions of eukaryotic, bacterial and archaeal eEF2 or EF-G sequences. Aligned residues for *Ct*Arg55 in switch 1 and the catalytic *Ct*His110 in switch 2 are boxed. While the histidine is universally conserved, the arginine finger is only present in eukaryotic eEF2. Eukaryotic and archaeal switch 1 are conserved in length, while bacterial effector loops are shorter and sequences diverged significantly. Sequence consensus (predominant shown nucleotide, X indicates parity) and overall conservation are indicated below the alignment. Identical nucleotides are color coded.

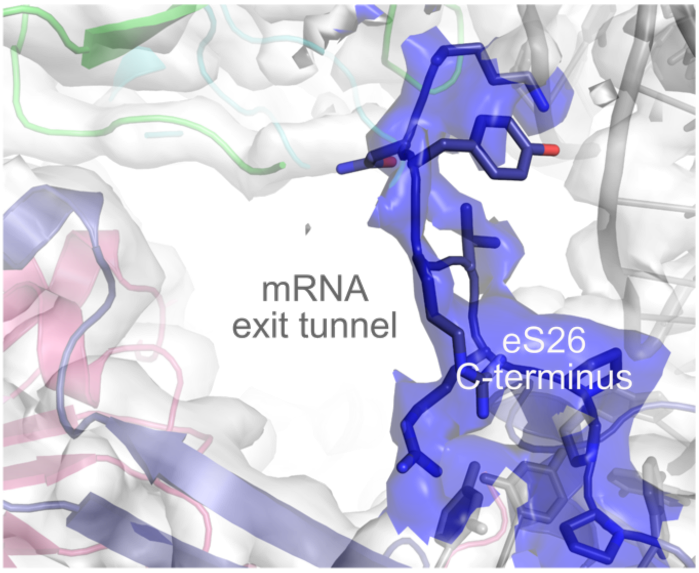

Supplementary Fig. 20 | Constriction of the mRNA exit tunnel. The mRNA exit tunnel of *Ct*80S is constricted by the C-terminus of eS26 (blue, in cryo-EM density at a 2.5 σ level).

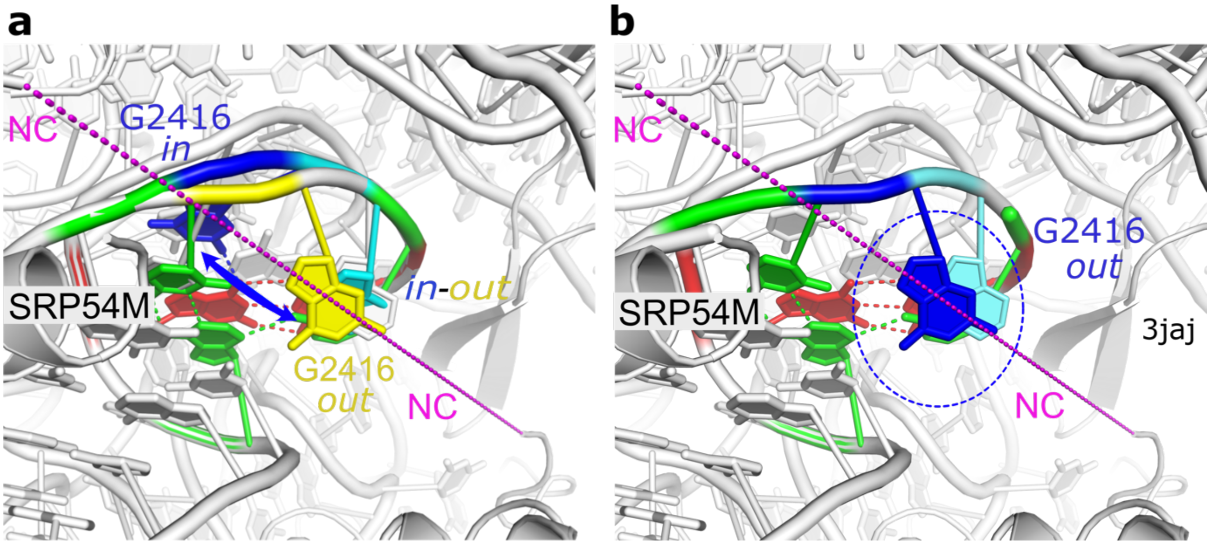

Supplementary Fig. 21 | A general mechanism for NC handover. **a**, **b**, Re-built cryo-EM models for a mammalian RNC in complex with SRP[7]. **a**, Superposition of the original model (3jaj) with G2416 flipped-in and our re-built model with G2416 bulged-out (yellow). Change is indicated by an arrow. **b**, Re-built model (according the EM-density) with bulged-out base (now blue, encircled) handing over the leaving NC (dashed line). Color coding and view as in Figure 3f.

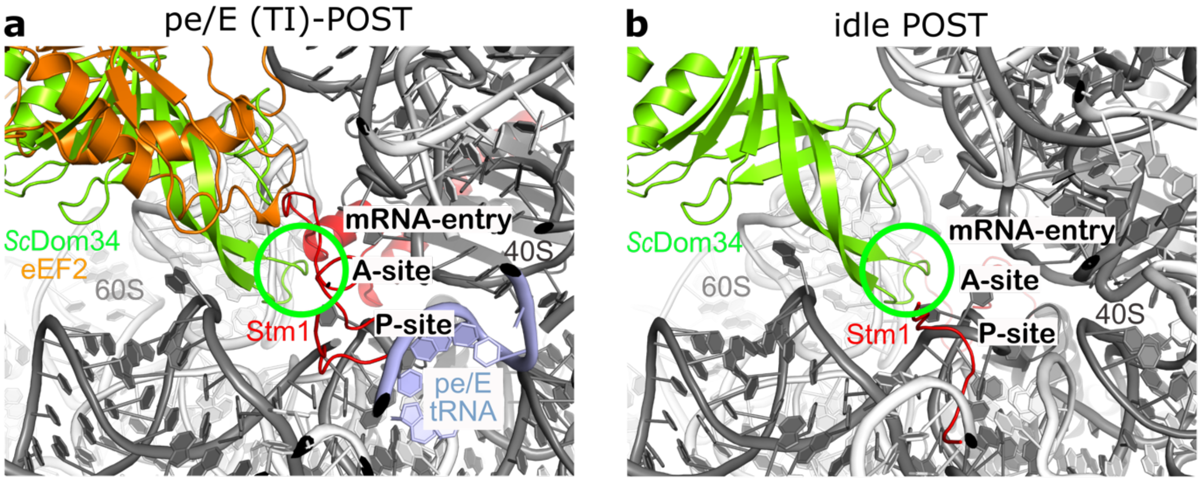

Supplementary Fig. 22 | Dom34 ribosomal recycling factor and Stm1. a, Superposition (based on 60S) of *Sc*Dom34 bound to *Sc*80S (PDB ID: 5m1j)[8] on the (TI)-POST state of *Ct*80S. *Sc*Dom34 interferes with Stm1 (and eEF2) binding in the A- and mRNA-entry sites in the rotated (TI)-POST state (indicated by green circle). **b**, Superposition of *Sc*Dom34 on the back-rotated idle POST state of *Ct*80S. The release of Stm1 (and eEF2) from the A- and mRNA-entry sites would allow for simultaneous binding of Dom34 and ribosome recycling.

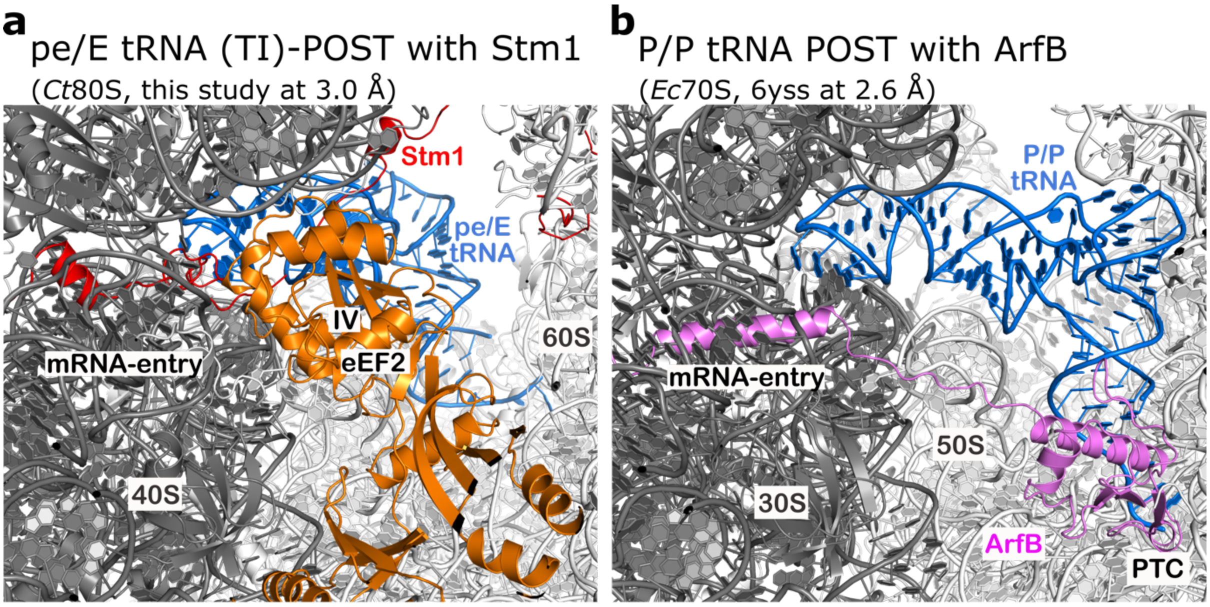

Supplementary Fig. 23 | ArfB ribosomal factor and Stm1 at the mRNA entry tunnel. Comparison of the Stm1 on the (TI)-POST state of *Ct*80S (**a**) and the *Ec*ArfB bound to *Ec*70S (PDB ID: 6yss)[9] (**b**). While Stm1 strictly follows the mRNA path backwards, ArfB fills the PTC with a folded domain and a GGQ-motif before entering the mRNA-entry tunnel backwards.

References

1. Natchiar, S.K., Myasnikov, A.G., Kratzat, H., Hazemann, I. & Klaholz, B.P. Visualization of chemical modifications in the human 80S ribosome structure. *Nature* **551**, 472-477 (2017).
2. Ben-Shem, A. et al. The structure of the eukaryotic ribosome at 3.0 A resolution. *Science* **334**, 1524-9 (2011).
3. Anger, A.M. et al. Structures of the human and Drosophila 80S ribosome. *Nature* **497**, 80-5 (2013).
4. Knorr, A.G. et al. Ribosome-NatA architecture reveals that rRNA expansion segments coordinate N-terminal acetylation. *Nat Struct Mol Biol* **26**, 35-39 (2019).
5. Pei, J. & Grishin, N.V. PROMALS3D: multiple protein sequence alignment enhanced with evolutionary and three-dimensional structural information. *Methods Mol Biol* **1079**, 263-71 (2014).
6. Watson, Z.L. et al. Structure of the bacterial ribosome at 2 A resolution. *Elife* **9**(2020).
7. Voorhees, R.M. & Hegde, R.S. Structures of the scanning and engaged states of the mammalian SRP-ribosome complex. *Elife* **4**(2015).
8. Hilal, T. et al. Structural insights into ribosomal rescue by Dom34 and Hbs1 at near-atomic resolution. *Nat Commun* **7**, 13521 (2016).
9. Chan, K.H. et al. Mechanism of ribosome rescue by alternative ribosome-rescue factor B. *Nat Commun* **11**, 4106 (2020).

Table 1. Data, refinement and model statistics

State	idle POST	pe/E tRNA (TI)-POST
Data collection statistics		
Microscope	Titan Krios	
Camera	K2	
Voltage (kV)	300	
Magnification	165,000	
Total dose ($e^-/Å^2$)	32.5	
Defocus rage (μm)	-0.8 to -2.0	
Calibrated pixel size (Å)	0.8127	
Micrographs collected	16,094	
Initial number of particles	727,659	
Refined particles	538,868	
Symmetry	C1	
Particles in final classes	279,818	35,338
Map resolution (Å)	2.9	3.0
Model Composition		
Chains	86	87
Atoms	207,345	214,210
Residues	Protein: 11,862<br>Nucleotide: 5,230	Protein: 12,678<br>Nucleotide: 5,307
Modifications	B8N: 1	B8N: 1
	OMG: 1	DDE: 1
	SAC: 1	
Ligands	ZN: 8	ZN: 8
	MG: 571	MG: 3
		GDP: 1
Water	945	0
Bonds (RMSD*)		
Length (Å) (# > 4σ)	0.003 (0)	0.004 (0)
Angles (°) (# > 4σ)	0.443 (8)	0.692 (81)
MolProbity score	1.51	1.91
Clash score	4.32	9.38
Ramachandran plot (%)		
Favored	95.66	93.76
Allowed	4.32	6.06
Outliers	0.03	0.18
Ramachandran plot Z-score (RMSD)		
Whole	-1.09 (0.07)	-2.11 (0.07)
Helix	-0.18 (0.08)	-0.08 (0.08)
Sheet	-0.18 (0.13)	-0.78 (0.12)
Loop	-1.19 (0.07)	-2.43 (0.06)
Rotamer outliers (%)	0.03	0.01
Cβ outliers (%)	0	0
Peptide plane (%)		
Cis proline/general	0.6/0.0	0.2/0.0
Twisted proline/general	0.0/0.0	0.2/0.0
CaBLAM outliers (%)	2.86	3.43
ADP min/max/mean ($Å^2$)		
Protein	57.66/437.43/123.53	70.62/373.99/148.19
Nucleotide	57.57/534.51/115.52	74.10/686.94/145.12
Ligand	53.49/616.94/90.54	102.55/242.29/158.71
Water	63.95/190.92/89.23	
Occupancy (%)	100	100
Model vs. Data (CC mask)	0.88	0.86

*RMSD: root-mean-square deviation

Table 2. Ribosomal proteins that are missing in or have mismatches with the *C. thermophilum* genome database.

Remark: The *C. globosum* sequences were used as template for modeling those *C. thermophilum* proteins where obvious discrepancies between cryo-EM density and the genome data base were noticed. These discrepencies are interpreted as data base errors as detailed below.

**Protein/Uniprot ID**	**Description**
uL6<br>Uniprot ID: G0S0E5	60S ribosomal protein l9-like protein [*Chaetomium thermophilum* var. *thermophilum* DSM 1495] NCBI Reference Sequence: XP_006691497.1.<br><br>The sequence modeled in the structure lacks 37 residues (16-52), but agrees with the sequences of *C. globosum* or *S. cerevisiae.*
eL19<br>Uniprot ID: G0S9T3	hypothetical protein CTHT_0047080 [*Chaetomium thermophilum* var. *thermophilum* DSM 1495] NCBI Reference Sequence: XP_006695079.1.<br><br>The sequence modeled in the structure agrees with this annotation, but starts from residue 2707.
eL24<br>Uniprot ID: G0S1P9	60S ribosomal protein L24-like protein [*Chaetomium thermophilum* var. *thermophilum* DSM 1495] NCBI Reference Sequence: XP_006691951.1.<br><br>The sequence modeled in the structure lacks 44 residues (18-61), but agrees with the sequences of *C. globosum* or *S. cerevisiae.*
uL15<br>Uniprot IDs:<br>G0SGT6 – *C. thermophilum*<br>Q2GXP7 – *C. globosum*	60S ribosomal protein L28-like protein [*Chaetomium thermophilum* var. *thermophilum* DSM 1495] NCBI Reference Sequence: XP_006697043.1.<br><br>60S ribosomal protein L28 [*Chaetomium globosum* CBS 148.51] NCBI Reference Sequence: XP_001224913.1.<br><br>In the structure the first 20 residues at the N-terminus are built as in *C. globosum*, and the remaining sequence (from residue 36) is built according *C. thermophilum*.
eL29<br>Uniprot ID:<br>Q2H9W4 – *C. globosum*	60S ribosomal protein L29 [*Chaetomium globosum* CBS 148.51] NCBI Reference Sequence: XP_001229506.1.<br><br>The protein is present in the *C. thermophilum* ribosome structure, but the sequence was not found in the *C. thermophilum* database. The sequence of *C. globosum* was modeled instead.
uL29<br>Uniprot ID: G0S0D7	ribosomal protein L35-like protein [*Chaetomium thermophilum* var. *thermophilum* DSM 1495] NCBI Reference Sequence: XP_006691489.1.<br><br>The sequence modeled in the structure lacks 809 residues (127-935), but agrees with the sequences of *C. globosum* or *S. cerevisiae.*

eL38<br>Uniprot ID: G0SG89	60S ribosomal protein L38-like protein [*Chaetomium thermophilum* var. *thermophilum* DSM 1495] NCBI Reference Sequence: XP_006696846.1.<br><br>The sequence modeled in the structure lacks 13 residues (23-35), but agrees with the sequences of *C. globosum* or *S. cerevisiae.*
eL39<br>Uniprot ID:<br>Q2H9R2 – *C. globosum*	60S ribosomal protein L39 [*Chaetomium globosum* CBS 148.51] NCBI Reference Sequence: XP_001229558.1.<br><br>The protein is present in the *C. thermophilum* ribosome structure, but the sequence was not found in the *C. thermophilum* database. The sequence of *C. globosum* was modeled instead.
eL40<br>Uniprot ID: G0S8G4	putative ribosomal protein [*Chaetomium thermophilum* var. *thermophilum* DSM 1495] NCBI Reference Sequence: XP_006693434.1.<br><br>The sequence modeled in the structure agrees with this annotation, but starts from residue 669.
eL41<br>Uniprot ID:<br>P0CX86 – *S. cerevisiae*	ribosomal 60S subunit protein L41A [*Saccharomyces cerevisiae* S288C] NCBI Reference Sequence: NP_010097.1.<br><br>The protein is present in the *C. thermophilum* ribosome structure, but the sequence was not found in the *C. thermophilum* or *C. globosum* databases. The sequence of *S. cerevisiae* was modeled instead.
uS14<br>Uniprot ID:<br>Q2HB68 – *C. globosum*	40S ribosomal protein S29 [Chaetomium globosum CBS 148.51] NCBI Reference Sequence: XP_001229052.1.<br><br>The protein is present in the *C. thermophilum* ribosome structure, but the sequence was not found in the *C. thermophilum* database. The sequence of *C. globosum* was modeled instead.

Table 3. Mass spectrometric analysis using intensity based absolute quantification (iBAQ) of the purified *Ct*80S.

Order	Protein	UniProt ID	Modifications	Coverage [%]	Peptides	iBAQ
1	uL4	G0SFC3	Met-loss [N-Term]; Met-loss+Acetyl [N-Term]	82	41	32.13
2	uS2	G0RYF9	Met-loss [N-Term]; Met-loss+Acetyl [N-Term]	74	22	30.65
3	eS1	G0S7T8		80	35	31.47
4	eS7	G0S8C4	Met-loss+Acetyl [N-Term]	74	20	31.75
5	uL16	G0SEI1		69	21	31.18
6	uS7	G0S1Z0	Met-loss [N-Term]; Met-loss+Acetyl [N-Term]	81	23	30.55
7	uL6	G0S0E5		63	23	30.53
8	eL6	G0S0D6	Met-loss+Acetyl [N-Term]	88	33	31.70
9	uS3	G0S6I7	Met-loss [N-Term]; Met-loss+Acetyl [N-Term]	89	30	31.51
10	eL8	G0SAJ9		81	40	31.58
11	eS4	G0S1A6		73	34	31.49
12	uL2	G0S4P8		74	20	30.95
13	uL22	G0SGY1	Met-loss [N-Term]; Met-loss+Acetyl [N-Term]	67	19	30.09
14	eL20	G0SCE2	Met-loss [N-Term]; Met-loss+Acetyl [N-Term]	72	21	31.65
15	uS4	G0S0Z4		63	22	31.33
16	uL30	G0SFL0	Met-loss [N-Term]; Met-loss+Acetyl [N-Term]	72	24	30.55
17	eS17	G0S871		66	15	30.83
18	uL11	G0SGH8		64	11	31.22
19	uL18	G0SEG2		79	36	31.88
20	uS9	G0SBR7	Met-loss [N-Term]; Met-loss+Acetyl [N-Term]	75	20	31.24
21	eL14	G0S1R0	Met-loss [N-Term]; Met-loss+Acetyl [N-Term]	69	18	31.49
22	eL13	G0S992	Met-loss [N-Term]; Met-loss+Acetyl [N-Term]	68	24	31.51
23	eL18	G0S9B5		56	18	30.11

24	uS11	G0SFL1		85	17	31.37
25	eS8	G0RY45		65	16	31.35
26	uL5	G0SHQ2	Met-loss+Acetyl [N-Term]	73	14	31.27
27	uL10	G0SGP3		59	24	31.43
28	uS8	G0SHI0	Met-loss [N-Term]; Met-loss+Acetyl [N-Term]	71	11	30.88
29	eL21	G0S0Z3		71	18	30.82
30	eL15	G0RZ88		45	14	30.11
31	RACK 1	G0S9U0	Met-loss+Acetyl [N-Term]	94	34	30.94
32	uS5	G0SAV4		67	24	30.78
33	uL15	G0SGT6		60	18	31.12
34	eL28	G0SHJ6	Met-loss [N-Term]; Met-loss+Acetyl [N-Term]	54	11	29.94
35	eS27	G0S1S4	Met-loss [N-Term]; Met-loss+Acetyl [N-Term]	51	5	31.65
36	uL3	G0RXW1		76	48	31.84
37	eL27	G0RZE9		71	15	31.04
38	uL23	G0S507		63	17	30.94
39	uS15	G0RZM9		59	11	30.54
40	eL33	G0SCL3		61	11	29.09
41	eS6	G0RY43	Met-loss [N-Term]	62	19	31.39
42	eL22	G0SDS8		57	12	29.88
43	eL19	G0S9T3		4	21	27.20
44	eL24	G0S1P9	Met-loss [N-Term]; Acetyl [N-Term]	37	15	30.87
45	uL1	G0S4Z9	Met-loss [N-Term]	61	20	30.61
46	P1	G0SEG9	Met-loss+Acetyl [N-Term]	85	11	32.69
47	uS13	G0S6J7		65	19	30.00
48	uL24	G0RYN9	Acetyl [N-Term]	64	17	31.81
49	eS10	G0RZD8		69	15	30.74
50	uS17	G0SF00	Met-loss [N-Term]; Met-loss+Acetyl [N-Term]	62	16	31.15
51	P2	G0S079		85	10	30.41
52	eS19	G0SFK8		71	17	31.38
53	eL32	G0S6V4		67	17	30.20
54	eL31	G0SD68		70	20	30.32
55	eL30	G0SEI0		73	12	31.32
56	eS28	G0S9M9	Acetyl [N-Term]	74	9	30.76
57	eS21	G0SI21	Acetyl [N-Term]	64	8	30.11

58	uL14	G0SI44		60	14	30.59
59	eS25	G0S1Y9	Met-loss [N-Term]	72	16	30.18
60	eS12	G0SHH9	Met-loss+Acetyl [N-Term]	90	22	31.00
61	uL13	G0SH61	Met-loss [N-Term]; Met-loss+Acetyl [N-Term]	54	18	30.33
62	uS10	G0S379		47	11	30.60
63	eL38	G0SG89	Met-loss [N-Term]; Met-loss+Acetyl [N-Term]	50	10	30.03
64	eS26	G0S2X4		55	9	30.90
65	uS19	G0S084	Met-loss+Acetyl [N-Term]	75	11	30.91
66	eEF2	G0S5T7	Met-loss [N-Term]; Met-loss+Acetyl [N-Term]	82	68	30.19
67	eS24	P0CU28	Met-loss [N-Term]; Met-loss+Acetyl [N-Term]	57	12	30.70
68	uS12	G0RY17		61	11	30.19
69	eL36	G0SBN1	Met-loss [N-Term]; Met-loss+Acetyl [N-Term]	43	7	28.00
70	uL29	G0S0D7		7	11	25.60
72	eL37	G0S101	Met-loss [N-Term]; Met-loss+Acetyl [N-Term]	45	8	29.66
74	eL43	G0S6B0		50	7	30.81
75	eS31	G0S2X5		64	14	30.01
76	Stm1	G0S428	Met-loss+Acetyl [N-Term]	76	36	29.50
77	eL34	G0SFN0		56	12	28.94
80	uS14	Q2HB68		14	2	27.70
81	eL42	G0SBZ6		42	9	28.60
217	eL29	Q2H9W4		37	3	29.96

Table 4. G+C and homoiteron content of the rRNAs from *C. thermophilum, C. globosum* and *S. cerevisiae.*

	C. thermophilum	***C. globosum***	***S. cerevisiae***
**26S rRNA** (G+C content)	52.3%	51.8%	47.9%
**18 S rRNA** (G+C content)	48.6%	48.7%	44.0%
**5.8 S rRNA** (G+C content)	48.7%	48.3%	45.5%
**5 S rRNA** (G+C content)	54.6%	50.8%	52.1%
**26S rRNA** (G homoiteron)	69	72	57
(C homoiteron)	42	37	22
**18S rRNA** (G homoiteron)	31	30	23
(C homoiteron)	19	17	10
**5.8S rRNA** (G homoiteron)	1	2	1
(C homoiteron)	1	2	1
**5S rRNA** (G homoiteron)	4	3	1
(C homoiteron)	3	3	1

Table 5. Inter-subunit bridges with 60S and 40S residues involved in the interactions.

	**40S subunit**		**60S subunit**	
**B1a**	No contact, uS19 N-ter not built due to its flexibility		No contact	
**B1b/c**	uS13	Asn10<br>Arg16<br>Asn21	uL5	Ile111<br>Lys116<br>Tyr117
**B2a**	18S	1639-1641<br>1754-1755<br>1776-1777<br>999-1000	26S	2227-2229<br>2219-2221
**B2b**	18S	992	26S	2158
**B2c**	18S	1120	26S	2154
**B2e**	18S	879-880	eL43	Arg85
**B3**	18S	1742-1746<br>1651-1653	26s	2264-2266<br>2253-2255<br>2088-2089<br>2292
			eL41	Arg21<br>Arg23
**B4**	18S	969-970<br>626-627	26S	828-829
**B5**	18S	1662-1663	26S	1914-1915
**B6**	18S	1720	eL24	Arg47
**B7a**	18S	911	26S	2170
	eS1	Lys13<br>Lys14		
**B7b/c**	18S	979-982	28S	1905-1907<br>832-834<br>2144 (through Mg^{2+} ion)
			eL43	Lys6<br>Lys7<br>Lys34
**B8 (only (TI)-POST state)**	18S	414<br>408-409	uL14	Ser133<br>Asn134<br>Asn30
**eB8**	eS1	Lys222<br>His132	26S	2499-2501
			No contact with eL8 (the C-ter is shorter than human eL8)	
**eB11**	eS8	Arg77<br>Asn87<br>Asn88<br>Glu89<br>Arg92<br>Arg125<br>Arg128<br>Arg168	26S	3285-3287<br>3292-3295<br>2069-2079<br>1922-1924
**eB12**	18S	847-852<br>811-813<br>855-856	eL19	Met159<br>Arg163<br>Arg165<br>Arg170

				Arg172<br>Gln174<br>Arg176<br>Gln177
**eB13**	eS6	Arg51<br>Val114<br>Lys131<br>Phe144<br>Phe145<br>Leu147<br>Asp150<br>Asp151<br>Tyr156<br>Ile158	eL24	Lys69-Ala85<br>Ile90<br>Arg93<br>Arg94<br>Arg101<br>Arg105<br>Ile109<br>Lys113<br>Arg116<br>Gln120<br>Lys123
	18S	161-163<br>1678-1681<br>1709-1710<br>1713		
**eB14**	18S	1767-1774<br>1110-1115<br>1122-1123<br>1639-1639<br>1649-50	eL41	Met1-Arg18<br>Ser24
