# Supplementary material for: High-resolution structures of a thermophilic eukaryotic 80S ribosome reveal atomistic details of translocation
Source: Nat Commun. 2022 Jan 25;13:476. doi: 10.1038/s41467-022-27967-9 (PMC8789840; doi:10.1038/s41467-022-27967-9)
Supplement: Supplementary file 2 — Reporting Summary [file 41467_2022_27967_MOESM2_ESM.pdf]

nature portfolio

Corresponding author(s): Irmgard Sinning

Last updated by author(s): Dec 17, 2021

Reporting Summary

Nature Portfolio wishes to improve the reproducibility of the work that we publish. This form provides structure for consistency and transparency in reporting. For further information on Nature Portfolio policies, see our Editorial Policies and the Editorial Policy Checklist.

Statistics

For all statistical analyses, confirm that the following items are present in the figure legend, table legend, main text, or Methods section.

n/a	Confirmed	
☐	☒	The exact sample size (n) for each experimental group/condition, given as a discrete number and unit of measurement
☐	☒	A statement on whether measurements were taken from distinct samples or whether the same sample was measured repeatedly
☒	☐	The statistical test(s) used AND whether they are one- or two-sided<br>*Only common tests should be described solely by name; describe more complex techniques in the Methods section.*
☒	☐	A description of all covariates tested
☐	☒	A description of any assumptions or corrections, such as tests of normality and adjustment for multiple comparisons
☒	☐	A full description of the statistical parameters including central tendency (e.g. means) or other basic estimates (e.g. regression coefficient) AND variation (e.g. standard deviation) or associated estimates of uncertainty (e.g. confidence intervals)
☒	☐	For null hypothesis testing, the test statistic (e.g. F, t, r) with confidence intervals, effect sizes, degrees of freedom and P value noted<br>*Give P values as exact values whenever suitable.*
☒	☐	For Bayesian analysis, information on the choice of priors and Markov chain Monte Carlo settings
☒	☐	For hierarchical and complex designs, identification of the appropriate level for tests and full reporting of outcomes
☒	☐	Estimates of effect sizes (e.g. Cohen's d, Pearson's r), indicating how they were calculated

Our web collection on statistics for biologists contains articles on many of the points above.

Software and code

Policy information about availability of computer code

Data collection	MassSpec: XCalibur 2.9 (Thermo Scientific); Cryo-EM: EPU 2 (Thermo Scientific)
Data analysis	MassSpec (iBAC): MaxQuant 1.6.12.0 (MPI Martinsried), Proteome discoverer 2.4 (Thermo Scientific), Perseus (MPI Martinsried); Cryo-EM: Cryo-EM images were processed with Relion 3.1, MotionCor2, CTFFIND4, and cisTEM 1.0.0. Molecular models were built with COOT 1.0 and refined using Phenix 1.19. Structures and cryo-EM densities were visualised and analyzed with UCSF Chimera 1.14, ChimeraX 1.0 and PyMOL 2.4.1 (Schrödinger, LLC).

For manuscripts utilizing custom algorithms or software that are central to the research but not yet described in published literature, software must be made available to editors and reviewers. We strongly encourage code deposition in a community repository (e.g. GitHub). See the Nature Portfolio guidelines for submitting code & software for further information.

Data

Policy information about availability of data

All manuscripts must include a data availability statement. This statement should provide the following information, where applicable:
- Accession codes, unique identifiers, or web links for publicly available datasets
- A description of any restrictions on data availability
- For clinical datasets or third party data, please ensure that the statement adheres to our policy

EM maps have been deposited in the Electron Microscopy Data Bank under accession codes EMDB: EMD-12976 for idle POST and EMDB: EMD-12977 for (TI)-POST conformational states.
The atomic models have been deposited in the Protein Data Bank under accession numbers PDB: 7OLC and PDB: 7OLD.
The mass spectrometry proteomics data have been deposited to the ProteomeXchange Consortium via the PRIDE [1] partner repository with the dataset identifier

PXD027370.
[1] Perez-Riverol Y et al. 2019, The PRIDE database and related tools and resources in 2019: improving support for quantification data. Nucleic Acids Res 47 (D1):D442-D450 (PubMed ID: 30395289).

Field-specific reporting

Please select the one below that is the best fit for your research. If you are not sure, read the appropriate sections before making your selection.

- [x] Life sciences
- [ ] Behavioural & social sciences
- [ ] Ecological, evolutionary & environmental sciences

For a reference copy of the document with all sections, see nature.com/documents/nr-reporting-summary-flat.pdf

Life sciences study design

All studies must disclose on these points even when the disclosure is negative.

Sample size	MassSpec: three independent samples; Cryo-EM: 727,659 cryo-EM micrographs were collected in total.
Data exclusions	MassSpec: no data exclusion; Cryo-EM: 188,791 cryo-EM micrographs were removed due to obvious faulty data collection (melted ice, contamination, too high defocus, low resolution after CTF estimation).
Replication	MassSpec: all attempts at replication were successful
Randomization	na
Blinding	na

Reporting for specific materials, systems and methods

We require information from authors about some types of materials, experimental systems and methods used in many studies. Here, indicate whether each material, system or method listed is relevant to your study. If you are not sure if a list item applies to your research, read the appropriate section before selecting a response.

Materials & experimental systems

n/a	Involved in the study
x	Antibodies
	x Eukaryotic cell lines
x	Palaeontology and archaeology
x	Animals and other organisms
x	Human research participants
x	Clinical data
x	Dual use research of concern

Methods

n/a	Involved in the study
x	ChIP-seq
x	Flow cytometry
x	MRI-based neuroimaging

Eukaryotic cell lines

Policy information about cell lines

Cell line source(s)	German collection of microorganisms and cell cultures (DSMZ)
Authentication	1495 (wild-type)
Mycoplasma contamination	tested negative
Commonly misidentified lines (See ICLAC register)	na
